# Fungal species associated with grapevine trunk diseases in Washington wine grapes and California table grapes, with novelties in the genera *Cadophora*, *Cytospora*, and *Sporocadus*

**DOI:** 10.3389/ffunb.2022.1018140

**Published:** 2022-10-07

**Authors:** Renaud Travadon, Daniel P. Lawrence, Michelle M. Moyer, Phillip T. Fujiyoshi, Kendra Baumgartner

**Affiliations:** ^1^ Department of Plant Pathology, University of California, Davis, Davis, CA, United States; ^2^ Department of Horticulture, Irrigated Agriculture Research and Extension Center, Washington State University, Prosser, WA, United States; ^3^ Crops Pathology and Genetics Research Unit, United States Department of Agriculture – Agricultural Research Service, Davis, CA, United States

**Keywords:** fungal ecology, fungal diversity, taxonomy, Sordariomycetes, Leotiomycetes, Diatrypaceae, Kalmusia, Thyrostroma

## Abstract

Grapevine trunk diseases cause serious economic losses to grape growers worldwide. The identification of the causal fungi is critical to implementing appropriate management strategies. Through a culture-based approach, we identified the fungal species composition associated with symptomatic grapevines from wine grapes in southeastern Washington and table grapes in the southern San Joaquin Valley of California, two regions with contrasting winter climates. Species were confirmed through molecular identification, sequencing two to six gene regions per isolate. Multilocus phylogenetic analyses were used to identify novel species. We identified 36 species from 112 isolates, with a combination of species that are new to science, are known causal fungi of grapevine trunk diseases, or are known causal fungi of diseases of other woody plants. The novel species *Cadophora columbiana*, *Cytospora macropycnidia*, *Cytospora yakimana*, and *Sporocadus incarnatus* are formally described and introduced, six species are newly reported from North America, and grape is reported as a new host for three species. Six species were shared between the two regions: *Cytospora viticola*, *Diatrype stigma*, *Diplodia seriata*, *Kalmusia variispora*, *Phaeoacremonium minimum*, and *Phaeomoniella chlamydospora*. Dominating the fungal community in Washington wine grape vineyards were species in the fungal families Diatrypaceae, Cytosporaceae and Sporocadaceae, whereas in California table grape vineyards, the dominant species were in the families Diatrypaceae, Togniniaceae, Phaeomoniellaceae and Hymenochaetaceae. Pathogenicity tests demonstrated that 10 isolates caused wood discoloration similar to symptomatic wood from which they were originally isolated. Growth rates at temperatures from 5 to 35°C of 10 isolates per region, suggest that adaptation to local climate might explain their distribution.

## Introduction

The numerous microbes associated with grapevine (*Vitis* sp.) play important roles in the plant’s nutrition and health status (Deyett and Rolshausen, 2020; Bettenfeld et al., 2022). Recent research efforts have characterized the grapevine microbiota in the rhizosphere, phyllosphere and endosphere (Bettenfeld et al., 2022; Cobos et al., 2022). The composition and structure of microbial communities have been found to be associated with vineyard management practices, vineyard location, and cultivars, suggesting that, for example, fruit and wine characteristics may be affected by the “microbial terroir” (Bokulich et al., 2014; Bokulich et al., 2016; Burns et al., 2016). The grapevine microbiota has also been investigated in relation to plant health (Bettenfeld et al., 2022), including its role relative to grapevine trunk diseases (Del Frari et al., 2019; Bruez et al., 2020; Pacetti et al., 2021; Patanita et al., 2022). These studies used culture-independent, DNA-metabarcoding approaches to reveal a considerable diversity of microbes associated with healthy and diseased grapevines, but these approaches have their limitations. First, studies of the fungal microbiota often rely on the sequencing of the ribosomal internal transcribed spacer (ITS) (Schoch et al., 2012), which may influence the results due to the specific PCR conditions and primers used (Jayawardena et al., 2018; Morales-Cruz et al., 2018b). Further, the high intra- and interspecific variability of the ITS region often limit the characterization of fungal communities to the genus level (Dissanayake et al., 2018). Second, the relationship between gene abundance and organismal abundance is not straightforward (Amend et al., 2010). While there are a combination of methods to confirm the viability of the taxa detected, such as metatranscriptomic analyses and/or complementary isolations on culture media (Eichmeier et al., 2018, Morales-Cruz et al., 2018a, Niem et al., 2020; Haidar et al., 2021; Nerva et al., 2022; Paolinelli et al., 2022; Vanga et al., 2022), fungi detected strictly through DNA sequencing may not be metabolically active (Baldrian et al., 2012). Most importantly, although extensive datasets are generated with these approaches, very little may be known about the biology and ecology of some taxa; the understanding of fungal communities cannot be achieved without understanding the ecology of individual species (Peay, 2014). Lastly, putatively novel species identified from metabarcoding studies, in the absence of physical specimens or cultures, cannot be properly nor formally characterized.

A traditional, field-based inventory of fungal biodiversity gathers the crucial details of taxonomy and life-history traits, which are required to test hypotheses on the roles of microbes in plant health, and importantly provides a tangible fungarium collection for sharing scientific knowledge that can last over time (Cazabonne et al., 2022). To describe fungal communities in grapevine, such culture-dependent approaches draw on mycological methods and rely on direct observations and microscopic examinations of diseased tissues and fungal structures, microbiological isolations, morphological characterizations of cultures and DNA-based phylogenetic analyses. The community composition is undoubtedly underestimated because artificial media and incubation conditions typically favor a subset of taxa (Jayawardena et al., 2018). Nonetheless, these approaches have enriched our understanding of fungal communities inhabiting grapevine as it relates to trunk diseases, either by comparing fungal community profiles over time (Kraus et al., 2019), among vines from different production areas (Úrbez-Torres et al., 2012), between symptomatic or asymptomatic plants (Hofstetter et al., 2012), or grown under different viticultural practices (Travadon et al., 2016). With physical culture collections, population genetics and genomics analyses can be conducted (Péros and Berger, 1999; Smetham et al., 2010; Travadon et al., 2012; Gramaje et al., 2013; Onetto et al., 2022), the pathogenicity of fungal isolates can be assessed experimentally (Cloete et al., 2015; Lawrence et al., 2015; Travadon and Baumgartner, 2015; Baloyi et al., 2018), interactions between species can be evaluated under controlled conditions (Lawrence et al., 2018b; Silva-Valderrama et al., 2021), and molecular databases including the ecological guild of species can be built (Lawrence et al., 2017b). Further, new pathogenic species can be described and linked with type specimens deposited in public repositories (Travadon et al., 2015; Lawrence et al., 2017a; Moyo et al., 2018; Kraus et al., 2020).

Grapevine trunk diseases (GTDs) are widespread throughout the world. The chronic wood infections cause a dieback and/or decline of vines, affecting fruit quality, reducing the productivity and longevity of vineyards, and causing serious economic losses to the viticulture industry (Kaplan et al., 2016; Sosnowski and Mccarthy, 2017; Baumgartner et al., 2019). GTDs encompass a set of diseases according to their etiology and fungal causal agents. The most common diseases are black foot disease (causal agents in the order Hypocreales and family Nectriaceae), Botryosphaeria dieback (Botryosphaeriales, Botryosphaeriaceae), Eutypa dieback (Xylariales, Diatrypaceae), esca and Petri disease (Helotiales, Ploettnerulaceae; Phaeomoniellales, Phaeomoniellaceae; Togniniales,Togniniaceae; Hymenochaetales, Hymenochaetaceae), and Phomopsis dieback (Diaporthales, Diaporthaceae). The causal agents are estimated to represent more than 130 fungal species (Gramaje et al., 2018), and they span four classes in the Pezizomycotina (Ascomycota) and 10 genera in the Hymenochaetales (Basidiomycota) (Lawrence et al., 2017b). Taxa belonging to the order Xylariales have recently been associated with dieback symptoms of grapevines (Lawrence et al., 2018b; Bahmani et al., 2021; Moghadam et al., 2022).

Among western North American viticulture regions, the fungal species causing grapevine trunk diseases have been examined primarily in California. Eleven species within the Diatrypaceae (Trouillas et al., 2010) and 15 species within the Botryosphaeriaceae (Úrbez-Torres et al., 2006; Úrbez-Torres, 2011; Rolshausen et al., 2013; Nouri et al., 2018) have been isolated from grapevine cankers, and their pathogenicity has been examined (Úrbez-Torres and Gubler, 2009; Trouillas and Gubler, 2010). Regarding the fungi associated with Petri disease and esca, *Cadophora luteo-olivacea*, *Cadophora melinii*, *Fomitiporia polymorpha*, *Inonotus vitis*, *Phaeoacremonium fraxinopennsylvanicum*, *Phaeoacremonium inflatipes*, *Phaeoacremonium minimum*, *Phaeoacremonium viticola*, and *Phaeomoniella chlamydospora* have all been isolated from symptomatic grapevines and are confirmed pathogenic (Scheck et al., 1998; Eskalen et al., 2005a; Eskalen et al., 2005b; Travadon et al., 2015; Brown et al., 2020). Additional esca and Petri disease pathogens identified in vineyards from British Columbia, Canada, include *Phaeoacremonium canadense*, *Phaeoacremonium iranianum*, and *Phaeoacremonium roseum* (Úrbez-Torres et al., 2014a). In California and British Columbia, Black foot pathogens include six and five species from the Nectriaceae, respectively, in the genera *Campylocarpon*, *Dactylonectria*, *Ilyonectria*, and *Thelonectria* (Úrbez-Torres et al., 2014b; Lawrence et al., 2019). In addition, seven species in the genus *Diaporthe* have been recovered from grapevines with dieback symptoms in California, and most species were confirmed pathogenic (Lawrence et al., 2015). Surveys of trunk diseases in vineyards in dry grape-growing regions of southern California (Coachella) and Mexico (Baja California and Sonora) report on species causing Botryosphaeria dieback, Eutypa dieback, and esca (Gispert et al., 2020; Úrbez-Torres et al., 2020; Rangel Montoya et al., 2021).

Important differences in environmental conditions and cultivar susceptibility may affect the species composition of grapevine trunk pathogens. The state of Washington is the second largest US producer of wine grapes and the largest producer of juice grapes in the US (National Agricultural Statistics Service, 2018). Wine grapes are grown principally in southeastern Washington, where the climate is a semi-arid steppe, characterized by very warm, dry summers, large diurnal temperature changes, and cold winters (USDA Cold Hardiness Zones 6-7), where injuries due to winter frost and freezing conditions are common. Most *Vitis vinifera* cultivars grown in Washington belong to the eco-geographic group *occidentalis*, which includes the small-berried wine grapes originating from western Europe (Aradhya et al., 2003). In Washington wine grape vineyards, Eutypa dieback can result in drastic yield reductions (Johnson and Lunden, 1987); however, the characterization of the pathogenic species is limited. To our knowledge, a single report listed the presence of *Cryptosphaeria pullmanensis*, *Diatrype whitmanensis*, *Eutypa laevata*, and *Eutypa lata* within the Diatrypaceae, *Diplodia* (*Dip*.) *seriata* and *Dip*. *mutila* within the Botryosphaeriaceae, *Cytospora chrysosperma* and *Cytospora rhodophila* within the Cytosporaceae, *Diaporthe eres* within the Diaporthaceae, and *Discostroma fuscellum* within the Amphisphaeriales [synonymized with Xylariales; (Holland et al., 2015)].

In California, table and raisin grapes are grown extensively in the southern San Joaquin Valley, which is characterized as a semi-arid climate with hot, dry summers and cool winters. The table grape cultivars belong to the eco-geographic group *orientalis* (large-berried table grapes of West Asia; e.g., ‘Thompson Seedless’, synonym ‘Sultanina’), *pontica* (intermediate type from the basin of the Black Sea and Eastern Europe; e.g., ‘Muscat of Alexandria’), and cultivars with mixed pedigree from these two groups (e.g., ‘Scarlet Royal’). Differences in susceptibility to vascular diseases among these three groups have been postulated (Travadon et al., 2013; Deyett et al., 2019). Reports of pathogenic species associated with trunk diseases in the southern San Joaquin Valley include members of the Botryosphaeriaceae, *Diaporthe ampelina* and *Eutypa lata* (Úrbez-Torres et al., 2006).

We investigated the fungal species cultured from the wood of grapevines with external symptoms of trunk diseases. To compare two western US regions with different climates and different cultivars, we surveyed 10 wine grape vineyards in Washington and 10 table grape vineyards in California, with an emphasis on vineyards with leaf symptoms of esca. We determined the pathogenicity to grapevine of a sub-sample of undescribed and under-studied species, and compared the thermophilic profiles of isolates from each region under controlled conditions, in order to assess if temperature could have an influence on the fungal community composition in each region. Finally, we described four species new to science.

## Material and methods

### Field sites and fungal isolations

In August 2018 and October 2018, we surveyed 10 wine grape vineyards in southeastern Washington State and 10 table grape vineyards in the southern San Joaquin Valley of California, respectively. The cooperation of local grape-growers and extension agents targeted vineyards with a history of trunk disease symptoms. Symptoms included general dieback symptoms, namely dead cordons, dead fruiting positions, and dead canes, usually present on vines affected by Botryosphaeria, Cytospora, Eutypa or Phomopsis diebacks. The selection of the 20 vineyards was also based on the presence of typical esca symptoms (interveinal leaf chlorosis and vine apoplexy observed in Summer) and typical Eutypa symptoms (stunted shoots, dwarfing and cupping of leaves with tattered margins observed in Spring). Sampled material consisted of diseased wood pieces from trunks and cordons. Internal symptoms revealed after cutting through the wood of symptomatic vines included vascular streaking, light brown to black discolorations, and white-rotted wood. Fifty-one wood samples were collected in Washington and 53 wood samples were collected in California. From each wood sample, 18 to 24 wood chips (4 × 4 × 2 mm) were cut from the margins of necrotic wood with flame-sterilized pruning shears, surface disinfected in 0.6% sodium hypochlorite (pH 7.2) for 30 s, rinsed in two serial baths of sterile deionized water for 30 s, dried on sterile filter paper and plated (6 to 8 wood chips per plate) either on potato dextrose agar (PDA, Difco, Detroit, MI) amended with tetracycline (1 mg/L) or malt extract agar (MEA, Difco) amended with streptomycin sulphate (100 mg/L) or water agar (Difco) amended with benomyl 50WP (4 mg/L) and streptomycin sulphate (100 mg/L). Incubation of Petri dishes occurred at 22°C in the dark for up to 28 days. Emerging colonies were hyphal-tip purified to PDA for further analyses.

### Fungal identification

Isolates were categorized into morphological groups based on colony morphology on PDA and limited microscopic observations. A preliminary species-level identification relied on the sequencing of the ITS gene region. Mycelium collection and DNA extraction followed the protocols outlined in Lawrence et al. (2018b). Fungi that are generally not considered as causal pathogens of trunk diseases were not examined further (62 isolates in the genera *Acremonium*, *Alternaria*, *Aureobasidium*, *Camarosporium*, *Collariella*, *Coniothyrium*, *Epicoccum*, *Fusarium*, *Peyronellea*, *Pseudocamarosporium*, *Trichoderma*, and *Tamaricicola*). For the remaining 112 isolates (**Table 1**), sequencing of the translation elongation factor 1 alpha (TEF-1α) region was performed for isolates in the families Botryosphaeriaceae, Cytosporaceae, Diaporthaceae, Didymosphaeriaceae, Dothidotthiaceae, Graphostromataceae, Hymenochaetaceae, Ploettnerulaceae, Physalacriaceae, Polyporaceae, Quambalariaceae, and Sporocadaceae. Sequencing of beta-tubulin (TUB2) was performed for isolates in the families Cytosporaceae, Diaporthaceae, Diatrypaceae, Phaeomoniellaceae, Ploettnerulaceae, Sporocadaceae, and Togniniaceae. The actin (ACT) gene region was sequenced for isolates in the families Cytosporaceae, Diaporthaceae, Dothidotthiaceae, and Togniniaceae. Sequencing of the second largest subunit of RNA polymerase II (RPB2) was performed for isolates in the families Cytosporaceae, Diaporthaceae, Dothidotthiaceae, Graphostromataceae, Hymenochaetaceae, Physalacriaceae, Polyporaceae, Quambalariaceae, and Sporocadaceae. Sequencing of the 28S ribosomal RNA gene (LSU) was performed for isolates in the families Cytosporaceae, Diaporthaceae, Physalacriaceae, Pleosporineae, Polyporaceae, and Sporocadaceae.

**Table 1:** List of isolates obtained from grapevine (*Vitis vinifera*) with wood symptoms of trunk diseases in California table grapes and Washington wine grapes, and associated GenBank accession numbers.

Family	Genus	Specific epithet	Isolate^1^	State^2^	County	Site	Cultivar	ITS	LSU	ACT	RPB2	TEF-1α	TUB
Botryosphaeriaceae	*Diplodia*	*seriata*	Kern802	CA	Kern	6	Autumn King	OP038084	–	–	–	OP106944	OP079902
Botryosphaeriaceae	*Lasiodiplodia*	*exigua*	Kern801	CA	Kern	2	Holiday	OP038083	–	–	–	OP106943	–
Botryosphaeriaceae	*Lasiodiplodia*	*gilanensis*	Kern803	CA	Kern	9	Autumn King	OP038085	–	–	–	OP106945	–
Botryosphaeriaceae	*Lasiodiplodia*	*gilanensis*	Kern804	CA	Kern	9	Autumn King	OP038086	–	–	–	OP106946	–
Didymosphaeriaceae	*Kalmusia*	*variispora*	Kern308	CA	Kern	5	Muscat	OP038056	–	–	–	OP106931	–
Didymosphaeriaceae	*Kalmusia*	*variispora*	Kern309	CA	Kern	5	Muscat	OP038057	–	–	–	OP106932	–
Didymosphaeriaceae	*Kalmusia*	*variispora*	Kern607	CA	Kern	7	Flame Seedless	OP038065	–	–	–	OP106939	–
Phaeomoniellaceae	*Phaeomoniella*	*chlamydospora*	Kern703	CA	Kern	3	Princess	OP038067	–	–	–	OP106940	OP079888
Phaeomoniellaceae	*Phaeomoniella*	*chlamydospora*	Kern707	CA	Kern	5	Muscat	OP038071	–	–	–	–	OP079892
Phaeomoniellaceae	*Phaeomoniella*	*chlamydospora*	Kern710	CA	Kern	7	Flame Seedless	OP038074	–	–	–	–	OP079895
Phaeomoniellaceae	*Phaeomoniella*	*chlamydospora*	Kern717	CA	Kern	7	Flame Seedless	OP038080	–	–	–	–	OP079899
Phaeomoniellaceae	*Phaeomoniella*	*chlamydospora*	Kern711	CA	Kern	7	Flame Seedless	OP038075	–	–	–	–	OP079896
Diaporthaceae	*Diaporthe*	*ampelina*	Kern903	CA	Kern	3	Princess	OP038090	OP076931	OP003973	OP095261	OP106950	OP079905
Diaporthaceae	*Diaporthe*	*ampelina*	Kern904	CA	Kern	3	Princess	OP038091	OP076932	OP003974	OP095262	OP106951	OP079906
Diaporthaceae	*Diaporthe*	*ampelina*	Kern902	CA	Kern	3	Princess	OP038089	OP076930	OP003972	OP095260	OP106949	OP079904
Diaporthaceae	*Diaporthe*	*ampelina*	Kern906	CA	Kern	7	Flame Seedless	OP038093	OP076934	OP003976	OP095264	OP106953	OP079908
Cytosporaceae	*Cytospora*	*macropycnidia*	Kern907 / CBS 149338	CA	Kern	10	Scarlet Royal	OP038094	OP076935	OP003977	OP095265	OP106954	OP079909
Cytosporaceae	*Cytospora*	*viticola*	Kern504	CA	Kern	5	Muscat	OM976604	ON059352	ON012557	ON045095	ON012571	ON086752
Cytosporaceae	*Cytospora*	*viticola*	Kern901	CA	Kern	3	Princess	OP038088	OP076929	OP003971	OP095259	OP106948	OP079903
Cytosporaceae	*Cytospora*	*viticola*	Kern905	CA	Kern	4	Crimson Seedless	OP038092	OP076933	OP003975	OP095263	OP106952	OP079907
Togniniaceae	*Phaeoacremonium*	*minimum*	Kern704	CA	Kern	5	Muscat	OP038068	–	OP003962	–	–	OP079889
Togniniaceae	*Phaeoacremonium*	*minimum*	Kern725	CA	Kern	5	Muscat	OP038082	–	OP003970	–	–	OP079901
Togniniaceae	*Phaeoacremonium*	*minimum*	Kern705	CA	Kern	5	Muscat	OP038069	–	OP003963	–	–	OP079890
Togniniaceae	*Phaeoacremonium*	*minimum*	Kern708	CA	Kern	5	Muscat	OP038072	–	OP003965	–	–	OP079893
Togniniaceae	*Phaeoacremonium*	*minimum*	Kern722	CA	Kern	7	Flame Seedless	OP038081	–	OP003969	–	–	OP079900
Togniniaceae	*Phaeoacremonium*	*minimum*	Kern709	CA	Kern	7	Flame Seedless	OP038073	–	OP003966	–	–	OP079894
Togniniaceae	*Phaeoacremonium*	*minimum*	Kern712	CA	Kern	7	Flame Seedless	OP038076	–	OP003967	–	–	OP079897
Togniniaceae	*Phaeoacremonium*	*minimum*	Kern714	CA	Kern	7	Flame Seedless	OP038077	–	OP003968	–	–	OP079898
Togniniaceae	*Phaeoacremonium*	*parasiticum*	Kern706	CA	Kern	5	Muscat	OP038070	–	OP003964	–	–	OP079891
Togniniaceae	*Phaeoacremonium*	*scolyti*	Kern701	CA	Kern	4	Crimson Seedless	OP038066	–	OP003961	–	–	OP079887
Diatrypaceae	*Cryptovalsa*	*ampelina*	Kern009	CA	Kern	10	Scarlet Royal	OP038052	–	–	–	–	OP079881
Diatrypaceae	*Diatrype*	*stigma*	Kern010	CA	Kern	10	Scarlet Royal	OP038053	–	–	–	–	OP079882
Diatrypaceae	*Diatrype*	*stigma*	Kern005	CA	Kern	1	Princess	OP038048	–	–	–	–	OP079878
Diatrypaceae	*Diatrype*	*stigma*	Kern306	CA	Kern	1	Princess	OP038054	–	–	–	–	OP079883
Diatrypaceae	*Diatrype*	*stigma*	Kern001	CA	Kern	2	Holiday	OP038044	–	–	–	–	OP079874
Diatrypaceae	*Diatrype*	*stigma*	Kern307	CA	Kern	2	Holiday	OP038055	–	–	–	–	OP079884
Diatrypaceae	*Diatrypella*	*verruciformis*	Kern002	CA	Kern	2	Holiday	OP038045	–	–	–	–	OP079875
Diatrypaceae	*Diatrypella*	*verruciformis*	Kern006	CA	Kern	2	Holiday	OP038049	–	–	–	–	OP079879
Diatrypaceae	*Eutypella*	*citricola*	Kern003	CA	Kern	4	Crimson Seedless	OP038046	–	–	–	–	OP079876
Diatrypaceae	*Eutypella*	*citricola*	Kern401	CA	Kern	4	Crimson Seedless	OP038063	–	–	–	–	OP079885
Diatrypaceae	*Eutypella*	*citricola*	Kern004	CA	Kern	4	Crimson Seedless	OP038047	–	–	–	–	OP079877
Diatrypaceae	*Eutypella*	*microtheca*	Kern008	CA	Kern	10	Scarlet Royal	OP038051	–	–	–	–	OP079880
Sporocadaceae	*Hyalotiella*	*transvalensis*	Kern605 / CBS 149294	CA	Kern	4	Crimson Seedless	OP038064	OP076926	OP003960	OP095255	OP106938	OP079886
Graphostromataceae	*Biscogniauxia*	*mediterranea*	Kern007	CA	Kern	8	Scarlet Royal	OP038050	–	–	OP095249	OP106930	–
Graphostromataceae	*Biscogniauxia*	*mediterranea*	Kern805	CA	Kern	9	Autumn King	OP038087	–	–	OP095258	OP106947	–
Hymenochaetaceae	*Fomitiporia*	*polymorpha*	Kern311	CA	Kern	7	Flame Seedless	OP038058	OP076921	–	OP095250	OP106933	–
Hymenochaetaceae	*Fomitiporia*	*polymorpha*	Kern312	CA	Kern	7	Flame Seedless	OP038059	OP076922	–	OP095251	OP106934	–
Hymenochaetaceae	*Fomitiporia*	*polymorpha*	Kern313	CA	Kern	7	Flame Seedless	OP038060	OP076923	–	OP095252	OP106935	–
Hymenochaetaceae	*Fomitiporia*	*polymorpha*	Kern314	CA	Kern	7	Flame Seedless	OP038061	OP076924	–	OP095253	OP106936	–
Hymenochaetaceae	*Fomitiporia*	*polymorpha*	Kern315	CA	Kern	7	Flame Seedless	OP038062	OP076925	–	OP095254	OP106937	–
Quambalariaceae	*Quambalaria*	*cyanescens*	Kern715	CA	Kern	6	Autumn King	OP038078	OP076927	–	OP095256	OP106941	–
Quambalariaceae	*Quambalaria*	*cyanescens*	Kern716	CA	Kern	6	Autumn King	OP038079	OP076928	–	OP095257	OP106942	–
Botryosphaeriaceae	*Diplodia*	*seriata*	Bent802	WA	Benton	1	Grenache / Sangiovese	OP038033	–	–	–	OP106920	OP079865
Botryosphaeriaceae	*Diplodia*	*seriata*	Bent803	WA	Benton	1	Grenache / Sangiovese	OP038034	–	–	–	OP106921	OP079866
Botryosphaeriaceae	*Diplodia*	*mutila*	Bent805	WA	Benton	3	Cabernet Sauvignon	OP038036	–	–	–	OP106923	–
Botryosphaeriaceae	*Diplodia*	*seriata*	Bent801	WA	Benton	1	Grenache / Sangiovese	OP038032	–	–	–	OP106919	–
Botryosphaeriaceae	*Dothiorella*	*iberica*	Bent804	WA	Benton	2	Chardonnay	OP038035	–	–	–	OP106922	–
Didymosphaeriaceae	*Kalmusia*	*variispora*	Bent603	WA	Yakima	10	Marsanne	OP038019	–	–	–	OP106913	–
Pleosporineae	*Thyrostroma*	*sp.*	Bent904	WA	Benton	2	Chardonnay	OP038037	OP076914	OP003954	OP095242	OP106924	OP079867
Phaeomoniellaceae	*Phaeomoniella*	*chlamydospora*	Bent708	WA	Benton	3	Cabernet Sauvignon	OP038021	–	–	–	–	OP079854
Phaeomoniellaceae	*Phaeomoniella*	*chlamydospora*	Bent710	WA	Benton	8	Chardonnay	OP038023	–	–	–	–	OP079856
Ploettnerulaceae	*Cadophora*	*columbiana*	Bent717 / CBS 149300	WA	Skamania	9	Pinot Noir	OP038026	–	–	–	OP106915	OP079859
Ploettnerulaceae	*Cadophora*	*columbiana*	Bent718 / CBS 149299	WA	Skamania	9	Pinot Noir	OP038027	–	–	–	OP106916	OP079860
Ploettnerulaceae	*Cadophora*	*ferruginea*	Bent721 / CBS 149295	WA	Grant	11	Grenache	OP038030	–	–	–	OP106917	OP079863
Ploettnerulaceae	*Cadophora*	*ferruginea*	Bent722	WA	Grant	11	Grenache	OP038031	–	–	–	OP106918	OP079864
Diaporthaceae	*Diaporthe*	*eres*	Bent909	WA	Skamania	9	Pinot Noir	OP038042	OP076919	OP003959	OP095247	OP106929	OP079872
Cytosporaceae	*Cytospora*	*yakimana*	Bent902 / CBS 149297	WA	Benton	5	Tinto Cao	OM976602	ON059350	ON012555	ON045093	ON012569	ON086750
Cytosporaceae	*Cytospora*	*yakimana*	Bent903 / CBS 149298	WA	Benton	5	Tinto Cao	OM976603	ON059351	ON012556	ON045094	ON012570	ON086751
Cytosporaceae	*Cytospora*	*viticola*	Bent503	WA	Benton	5	Tinto Cao	OP038011	OP076904	OP003949	OP095232	OP106912	OP079846
Cytosporaceae	*Cytospora*	*viticola*	Bent901 / CBS 149296	WA	Benton	5	Souzao	OM976601	ON059349	ON012554	ON045092	ON012568	ON086749
Cytosporaceae	*Cytospora*	*viticola*	Bent907	WA	Benton	8	Chardonnay	OP038040	OP076917	OP003957	OP095245	OP106927	OP079870
Cytosporaceae	*Cytospora*	*viticola*	Bent908	WA	Benton	8	Chardonnay	OP038041	OP076918	OP003958	OP095246	OP106928	OP079871
Cytosporaceae	*Cytospora*	*viticola*	Bent411	WA	Benton	8	Chardonnay	OP038010	OP076903	OP003948	OP095231	OP106911	OP079845
Cytosporaceae	*Cytospora*	*viticola*	Bent401	WA	Benton	8	Chardonnay	OM976600	ON059348	ON012553	ON045091	ON012567	ON086748
Cytosporaceae	*Cytospora*	*viticola*	Bent905	WA	Benton	8	Riesling	OP038038	OP076915	OP003955	OP095243	OP106925	OP079868
Cytosporaceae	*Cytospora*	*viticola*	Bent906	WA	Benton	8	Riesling	OP038039	OP076916	OP003956	OP095244	OP106926	OP079869
Togniniaceae	*Phaeoacremonium*	*minimum*	Bent719	WA	Grant	11	Grenache	OP038028	–	OP003952	–	–	OP079861
Togniniaceae	*Phaeoacremonium*	*minimum*	Bent720	WA	Grant	11	Grenache	OP038029	–	OP003953	–	–	OP079862
Togniniaceae	*Phaeoacremonium*	*minimum*	Bent709	WA	Benton	8	Chardonnay	OP038022	–	OP003950	–	–	OP079855
Togniniaceae	*Phaeoacremonium*	*minimum*	Bent712	WA	Benton	8	Chardonnay	OP038024	–	OP003951	–	–	OP079857
Diatrypaceae	*Diatrype*	*stigma*	Bent015	WA	Benton	1	Grenache / Sangiovese	OP038001	–	–	–	–	OP079837
Diatrypaceae	*Diatrype*	*whitmanensis*	Bent019	WA	Grant	11	Grenache	OP038005	–	–	–	–	OP079840
Diatrypaceae	*Diatrype*	*whitmanensis*	Bent020	WA	Grant	11	Grenache	OP038006	–	–	–	–	OP079841
Diatrypaceae	*Diatrype*	*whitmanensis*	Bent023	WA	Grant	11	Grenache	OP038009	–	–	–	–	OP079844
Diatrypaceae	*Diatrype*	*whitmanensis*	Bent002	WA	Benton	1	Grenache / Sangiovese	OP037989	–	–	–	–	OP079828
Diatrypaceae	*Diatrype*	*whitmanensis*	Bent004	WA	Benton	3	Cabernet Sauvignon	OP037991	–	–	–	–	OP079830
Diatrypaceae	*Eutypa*	*cerasi*	Bent001	WA	Benton	2	Chardonnay	OP037988	–	–	–	–	OP079827
Diatrypaceae	*Eutypa*	*lata*	Bent021	WA	Grant	11	Grenache	OP038007	–	–	–	–	OP079842
Diatrypaceae	*Eutypa*	*lata*	Bent022	WA	Grant	11	Grenache	OP038008	–	–	–	–	OP079843
Diatrypaceae	*Eutypa*	*lata*	Bent003	WA	Benton	1	Grenache / Sangiovese	OP037990	–	–	–	–	OP079829
Diatrypaceae	*Eutypa*	*lata*	Bent009	WA	Benton	6	Cabernet Sauvignon	OP037995	–	–	–	–	OP079831
Diatrypaceae	*Eutypa*	*lata*	Bent010	WA	Benton	6	Cabernet Sauvignon	OP037996	–	–	–	–	OP079832
Diatrypaceae	*Eutypa*	*lata*	Bent011	WA	Benton	6	Cabernet Sauvignon	OP037997	–	–	–	–	OP079833
Diatrypaceae	*Eutypa*	*lata*	Bent012	WA	Benton	6	Cabernet Sauvignon	OP037998	–	–	–	–	OP079834
Diatrypaceae	*Eutypa*	*petrakii* var *hederae*	Bent014	WA	Benton	8	Chardonnay	OP038000	OP076901	–	–	–	OP079836
Diatrypaceae	*Eutypa*	*petrakii* var *hederae*	Bent013	WA	Benton	8	Chardonnay	OP037999	OP076900	–	–	–	OP079835
Diatrypaceae	*Eutypella*	*virescens*	Bent017	WA	Grant	11	Grenache	OP038003	–	–	–	–	OP079838
Diatrypaceae	*Eutypella*	*virescens*	Bent018	WA	Grant	11	Grenache	OP038004	–	–	–	–	OP079839
Sporocadaceae	*Sporocadus*	*kurdistanicus*	Bent506 / CBS 149336	WA	Benton	2	Chardonnay	OP038014	OP076907	–	OP095235	–	OP079849
Sporocadaceae	*Sporocadus*	*kurdistanicus*	Bent504	WA	Benton	3	Cabernet Sauvignon	OP038012	OP076905	–	OP095233	–	OP079847
Sporocadaceae	*Sporocadus*	*kurdistanicus*	Bent507	WA	Benton	3	Cabernet Sauvignon	OP038015	OP076908	–	OP095236	–	OP079850
Sporocadaceae	*Sporocadus*	*kurdistanicus*	Bent505	WA	Benton	3	Cabernet Sauvignon	OP038013	OP076906	–	OP095234	–	OP079848
Sporocadaceae	*Sporocadus*	*kurdistanicus*	Bent509	WA	Benton	8	Chardonnay	OP038016	OP076909	–	OP095237	–	OP079851
Sporocadaceae	*Sporocadus*	*kurdistanicus*	Bent510	WA	Benton	8	Riesling	OP038017	OP076910	–	OP095238	–	OP079852
Sporocadaceae	*Sporocadus*	*kurdistanicus*	Bent910	WA	Benton	8	Riesling	OP038043	OP076920	–	OP095248	–	OP079873
Sporocadaceae	*Sporocadus*	*kurdistanicus*	Bent511	WA	Benton	8	Riesling	OP038018	OP076911	–	OP095239	–	OP079853
Sporocadaceae	*Sporocadus*	*incarnatus*	Bent716 / CBS 149301	WA	Benton	8	Riesling	OP038025	OP076913	–	OP095241	–	OP079858
Physalacriaceae	*Flammulina*	*filiformis*	Bent016	WA	Benton	1	Grenache / Sangiovese	OP038002	OP076902	–	OP095230	OP106910	–
Physalacriaceae	*Flammulina*	*filiformis*	Bent006	WA	Benton	6	Syrah	OP037992	OP076897	–	OP095227	OP106907	–
Physalacriaceae	*Flammulina*	*filiformis*	Bent007	WA	Benton	6	Syrah	OP037993	OP076898	–	OP095228	OP106908	–
Physalacriaceae	*Flammulina*	*filiformis*	Bent008	WA	Benton	6	Syrah	OP037994	OP076899	–	OP095229	OP106909	–
Polyporaceae	*Trametes*	*versicolor*	Bent701	WA	Benton	5	Tinto Cao	OP038020	OP076912	–	OP095240	OP106914	–

^1^ Kern isolates originate from California and Bent isolates originate from Washington state. Kern and Bent isolates are maintained in the culture collection of Dr. Kendra Baumgartner, USDA-ARS laboratory, UC Davis. CBS: Culture collection of the Westerdijk Fungal Biodiversity Institute, Utrecht, The Netherlands.

^2^ CA: California, isolates collected in October 2018; WA: Washington, isolates collected in August 2018.

DNA samples were used undiluted as template in PCR, or diluted 1:10 when the undiluted template yielded no product. Amplifications were performed in 25 μL reactions containing 1× GoTaq® Flexi colorless buffer (Promega, Madison, Wisconsin, USA), 1 μM of each primer and 1 μL DNA (**Supplementary Table 1**). Magnesium concentrations were titrated to 3 mM for Actin for greater primer specificity. All extension steps were at 72°C. Conditions for some primer pairs were made more stringent for difficult templates or were altered slightly for efficient thermocycler use. Successful amplifications were verified by gel electrophoresis and bidirectionally sequenced by the UC Davis College of Biological Sciences DNA Sequencing Facility.

Forward and reverse nucleotide sequences were assembled, proofread, and edited in Sequencher 5.4.6 (Gene Codes Corporation, Ann Arbor, Michigan, USA), and deposited in GenBank (**Table 1**). Preliminary species identities were obtained by BLASTn searches of sequences against the nucleotide database of GenBank from the National Center for Biotechnology Information (NCBI) and the curated molecular repository of grapevine trunk pathogens TrunkDiseaseID.org (Lawrence et al., 2017b). The sequences of some of the isolates did not match any known molecular data in either database. In these cases, sequences with high similarity from ex-type and non-type isolates were included for phylogenetic reference utilizing the BLASTn function in NCBI and extensive literature review. Accordingly, relevant reference sequences were retrieved from recent and comprehensive phylogenetic studies for isolates in the genus *Cadophora* [(Travadon et al., 2015; Linnakoski et al., 2018; Walsh et al., 2018; Bien and Damm, 2020; Maciá-Vicente et al., 2020; Aigoun-Mouhous et al., 2021; Chen et al., 2022; Koukol and Maciá-Vicente, 2022); **Supplementary Table 2**], *Cytospora* [(Lawrence et al., 2018a; Pan et al., 2020; Shang et al., 2020; Pan et al., 2021); **Supplementary Table 3**], and *Sporocadus* [(Liu et al., 2019; Bundhun et al., 2021; Moghadam et al., 2022); **Supplementary Table 4**].

Multiple sequence alignments were performed in MEGA v. 6 (Tamura et al., 2013) and manually adjusted where necessary in Mesquite v. 3.10 (Maddison and Maddison, 2016). Phylogenetic analyses were performed for concatenated datasets. Each dataset was analyzed using two different optimality search criteria, maximum parsimony (MP) and maximum likelihood (ML), in PAUP* v. 4.0a169 and GARLI v. 0.951, respectively (Swofford, 2002; Zwickl, 2006). For MP analyses, heuristic searches with 1000 random sequence additions were implemented with the Tree-Bisection-Reconnection algorithm; gaps were treated as missing data. Bootstrap analyses with 1000 pseudoreplicates were used to estimate branch support. For ML analyses, MEGA was used to infer a model of nucleotide substitution for each dataset, using the Akaike Information Criterion (AIC). All ML analyses were conducted using the best-fit model of nucleotide substitution using the default parameters in GARLI. Branch support was determined by 1000 bootstrap pseudoreplicates. Sequences of *Hyaloscypha finlandica*, *Diaporthe ampelina*, and *Seimatosporium luteosporum* and *Seimatosporium pistaciae* served as the outgroup taxa in phylogenetic analyses of the genus *Cadophora*, *Cytospora*, and *Sporocadus*, respectively.

### Morphological characterization of putative new species

For all the morphological descriptions, colony colors were assessed following Rayner (1970) and photographs were obtained using either a Leica DM500B (Leica microsystems CMS, Wetzlar, Germany) light microscope equipped with a Leica color video camera (model DFC 310 FX) or a Olympus CX31 (Olympus Corporation, Tokyo, Japan) light microscope equipped with a ToupTek Photonics camera (model E3ISPM Series C-mount USB3.0 CMOS; ToupTek Photonics Co., Ltd., Hangzhou, China). For macroscopic features, a binocular Wild Heerbrugg M8 (Leica microsystems CMS) stereomicroscope equipped with an identical ToupTek Photonics camera was used. All measurements were made with the software ToupView (ToupTek Photonics Co., Ltd.). Thirty measurements were made for all the observed micro-morphological structures and results are presented as min-(average)-max.

Isolates of putative new species in the genus *Cadophora* were characterized morphologically according to Travadon et al. (2015). Colony growth and characters were obtained on 2% MEA and 3.9% PDA media at 22°C in the dark. Microscopic slides were produced by mounting vegetative hyphae, conidia, conidiogenous cells, collarettes and conidiophores in water or in 50% glycerol and measurements were obtained at 1000× magnification. Isolates of putative new species in the genus *Cytospora* were characterized morphologically according to Lawrence et al. (2018a). Colony growth and characters were obtained on 2% PDA medium after 4 to 21 days at 22°C in the dark. Pycnidia were induced on grapevine wood embedded in PDA as detailed by Lawrence et al. (2017a). Pycnidial squash mounts were obtained in 50% glycerol and measurements of vegetative hyphae, conidia, and conidiogenous cells were obtained at 1000× magnification. Pycnidia characterization (diameter, presence/absence of a conceptacle, and color) was achieved with a binocular dissecting microscope. For isolates of putative new species in the genus *Sporocadus*, colony descriptions and microscopic structures were obtained from cultures on 3.9% PDA and 2% MEA. Conidiomata, mycelium, conidiophores, conidiogenous cells and conidia were measured.

### Pathogenicity tests

In a replicated greenhouse trial conducted in 2019, ten fungal isolates (Bent708, Bent901, Bent718, Bent603, Bent008, Bent904, Bent721, Kern004, Kern007, Bent902), representative of ten distinct species identified by molecular analyses (**Table 1**), were selected for inoculations to the woody stems of potted *Vitis vinifera* ‘Chardonnay’. For four isolates that did not produce spores readily in culture (Bent008, Bent904, Kern004, and Kern007), mycelial suspensions were prepared as inoculum [5-day-old cultures grown in Potato Dextrose Broth (PDB) and homogenized with a hand-held disperser], following the protocol outlined in Travadon et al. (2013). Conidial suspensions served as inoculum for the six remaining isolates, with all inoculum adjusted to 1 × 10^5^ spores or mycelial fragments/mL using a hemacytometer.

In the pathogenicity test, two replicate experiments were performed, starting two weeks apart, on two sets of plants propagated in two separate greenhouses. In each experiment, 32 plants inoculated with water served as control plants and 32 plants inoculated with conidia of *Phaeomoniella chlamydospora* isolate Bent708 served as positive controls (64 plants × two experiments = 128 plants). For the other nine isolates, 16 plants were inoculated with each isolate per experiment (nine inoculation treatments × 16 plants × two experiments = 288 plants). The plants were arranged in a completely randomized design in each greenhouse. Plants were propagated from dormant cuttings according to Travadon et al. (2013). Briefly, starting in May 2019, cuttings were callused at 30°C and 100% humidity in a mixture of perlite and vermiculite (1:1, vol/vol) for 21 days. Once shoot and root initials emerged from the callus tissue, cuttings were coated with melted paraffin wax (Gulf Wax; Royal Oak Enterprises, L.L.C, Roswell, GA, USA) to prevent moisture loss and transplanted into pots containing sterile potting mix amended with slow-release fertilizer (Osmocote® Pro 24-4-9, Scotts, Marysville, Ohio, USA). Plants were grown in the greenhouse at the University of California Experiment Station in Davis [natural sunlight photoperiod, 25 ± 1°C (day), 18 ± 3°C (night)], with some modifications to the temperature conditions [10 ± 2°C (day), 4 ± 2°C (night)] during dormancy (from December to February). During the growing season, plants were watered four times per week for 10 min using a drip-irrigation system (0.5 L h-1). Approximately 2 months after being transplanted in September 2019, the woody stem of each grapevine was wounded (2 mm-width × 3 mm-depth) with a power drill to produce a wound approximately 2 cm below the uppermost node. Inoculum (20 µl) was pipetted into the wound, which was then sealed with Vaseline (Unilever, Rotterdam, London, UK) and Parafilm (Bemis Co., Neenah, Wisconsin, USA) to prevent inoculum desiccation. Non-inoculated controls were wounded and ‘mock-inoculated’ with sterile water.

As inoculated plants did not develop foliar symptoms of trunk diseases during the 12-month incubation period, we used as a measure of pathogenicity the length of wood discoloration (LWD) extending from the inoculation site. To reveal these wood lesions, the green shoots, roots, and bark of each plant were removed with a flame sterilized knife or pruning shears. The woody stems were surface disinfected in 1% sodium hypochlorite for 2 min and rinsed with deionized water. The length of each stem was recorded and cut longitudinally through the inoculation site to expose wood discoloration, the length of which was measured with a digital caliper. To confirm that the pathogen was responsible for wood discoloration in inoculated plants, recovery was attempted by cutting 10 pieces (2 × 5 × 5 mm) of wood from the distal margin of the lesion, followed by surface disinfestation in 0.6% sodium hypochlorite (pH 7.2) for 30 s, two 30-s rinses in sterile deionized water, plating on PDA amended with tetracycline (1 mg L-1), and incubation in the dark at approximately 22°C for 14 to 21 days.

Normality and homogeneity of variances were evaluated before an analysis of variance (ANOVA) was used to determine the effect of each isolate on LWD. The ANOVA was performed using the MIXED procedure in SAS, with experiment considered as a random effect. Means were calculated using the LS-Means procedure. Pairwise mean differences in LWD between inoculated and mock-inoculated plants were analyzed using Dunnett’s tests (*P*< 0.05). The recovery of isolates from inoculated plants served to complete Koch’s postulates and, as such, was considered a second measure of pathogenicity. Recovery rates were estimated as the number of plants from which the isolate was recovered out of the total number of plants inoculated.

### Effect of temperature on mycelial growth

Ten isolates from each geographical region were assessed *in vitro* for their optimal growth temperature. The ten isolates from Washington represented six species and the ten isolates from California represented eight species. All isolates were cultured on 3.9% PDA, except for isolates of the genus *Cadophora*, which were cultured on 2% MEA. Optimal growth temperature was tested by culturing each isolate in triplicate in the dark at temperatures ranging from 5°C to 35°C at 5°C increments. Mycelial plugs (5mm diam.) were taken from the margin of an actively growing culture and transferred to the center of 90-mm diam. dishes. Radial growth was measured from two to 14 days after inoculations, depending on how fast each isolate’s mycelium grew in culture (e.g., 2 days for *Biscogniauxia mediterranea* and 14 days for *Phaeoacremonium scolyti*). Two perpendicular measurements were taken of the colony diameter. Each experiment was conducted twice. For each isolate, the average colony diameters at each temperature were adjusted to a non-linear regression curve to estimate their optimal growth temperature using the program SigmaPlot v. 14.0 (Systat Software Inc., San Jose, California). For this purpose, data were assumed to follow a Gaussian function of the form:


$$Y=ae^{[-0.5}(\frac{x\;–\;x0}{b}{)}^{2}]$$

With *a* the height of the curve’s peak, *x_0_* the position of the center of the peak (i.e., optimal temperature) and *b* the Gaussian root mean square controlling the width of the curve. For each isolate, normality of data distribution was tested with the Shapiro-Wilk test and homoscedasticity was tested by computing the Spearman rank correlation between the absolute values of the residuals and the observed value of the dependent variable (α = 0.05). Goodness of fit was evaluated through the computation of the coefficient of determination *R* ^2^.

## Results

### Vineyard sampling and symptom observations

Of the 10 wine grape vineyards in Washington, five sites (sites 1, 2, 5, 9 and 11; see **Table 1** for locations and cultivars) had typical leaf symptoms of esca (interveinal discolorations and scorching), with apoplectic vines (severe form of esca) also present at sites 5 and 9. Among the five sites with esca symptoms, cross-sections through trunks and cordons revealed black spots (sometimes present in concentric rings in the wood) at all five sites, with white-rotted wood also present at sites 1, 5 and 11. Among the Washington sites with general dieback symptoms (dead spur positions and/or entire dead cordons at sites 6, 8 and 10) cross-sections through trunks and cordons revealed dark brown to black wood cankers at all three sites. White-rotted wood was observed at sites 1, 5 and 11. Leaf symptoms of grapevine leaf roll viruses were present at site 8. Leaf symptoms of Eutypa dieback were present at site 3. Pathogenic species were isolated from the nine Washington sites with trunk disease symptoms, with up to six species isolated from site 1 (**Table 1**). In contrast, one Washington site (site 7) had peculiar canopy symptoms with uncommon leaf scorching, black spots on shoots, petioles swollen at the base, and vines with wilted and dead cordons; isolation attempts from wood samples did not yield any fungal pathogens.

From the 10 table grape vineyards in California, seven sites (sites 1, 2, 3, 5, 6, 7, and 8) had typical leaf symptoms of esca, with apoplectic vines also present at site 7. Wood symptoms in these seven sites included both black spotting and wood discolorations, varying in color from black to brown to pinkish. General dieback symptoms were present at sites 1, 2, 3, 4, 7, 9 and 10. Wood symptoms at site 10 were extensive brown lesions present in young cordons, whereas wood symptoms at site 7 were exclusively black spots (sometimes present in concentric rings). There were four sites (sites 1, 2, 3 and 7) with symptoms of both esca and dieback. Pathogenic species were isolated from all 10 vineyards.

### Identification of isolates

Molecular identification based on the sequencing of two to six loci per isolate, and subsequent sequence comparisons with type/voucher specimens, identified 112 isolates of 36 fungal species either frequently associated with grapevine trunk diseases or known causal agents of dieback in other woody plants. Sixty isolates from Washington represented 22 species whereas 52 isolates from California represented 20 species (**Table 1**). The following six species were shared between Washington and California: *Cytospora viticola*, *Diatrype stigma*, *Diplodia seriata*, *Kalmusia variispora*, *Phaeoacremonium minimum*, and *Phaeomoniella chlamydospora*.

Dominating the community of pathogenic species in California table grape vineyards (61.5% of California isolates) were species in the families Diatrypaceae (12 isolates of five species: *Cryptovalsa ampelina*, *Diatrype stigma*, *Diatrypella verruciformis*, *Eutypella citricola* and *Eutypella microtheca*), Togniniaceae (10 isolates of three species: *Phaeoacremonium minimum*, *Phaeoacremonium parasiticum* and *Phaeoacremonium scolyti*), Phaeomoniellaceae (five isolates of *Phaeomoniella chlamydospora*) and Hymenochaetaceae (five isolates of *Fomitiporia polymorpha*). Other known pathogenic species included four isolates from the family Botryosphaeriaceae (*Diplodia seriata*, *Lasiodiplodia exigua* and *Lasiodiplodia gilanensis*) and eight isolates from the order Diaporthales (*Diaporthe ampelina*, *Cytospora viticola* and a second *Cytospora* species whose sequences did not match any known species in molecular databases). From the 112 total isolates recovered from the two regions, two isolates from the genus *Cadophora*, three isolates from the genus *Cytospora*, and one isolate from the genus *Sporocadus* did not have any affiliations with type or non-type specimens in GenBank; hence, multilocus phylogenetic analyses were conducted to circumscribe these undescribed taxa.

Dominating the community of pathogenic species in Washington wine grape vineyards (61.7% of Washington isolates) were species in the fungal families Diatrypaceae (18 isolates of six species), Cytosporaceae (10 isolates of two *Cytospora* species) and Sporocadaceae (nine isolates of two species). Fungal species in the Diatrypaceae included *Diatrype stigma*, *Diatrype whitmanensis*, *Eutypa cerasi*, *Eutypa lata*, *Eutypa petrakii* var *hederae*, and *Eutypella virescens*. Pathogenic species often associated with esca included *Phaeomoniella chlamydospora* and *Phaeoacremonium minimum* in addition to Basidiomycetes *Flammulina filiformis* and *Trametes versicolor*. Two species of *Cadophora* were also recovered. Only five isolates of the family Botryosphaeriaceae (*Diplodia seriata*, *Diplodia mutila* and *Dothiorella iberica*) were isolated from Washington sites.

### Phylogenetic analyses of the genus *Cadophora*

**Figure 1:**
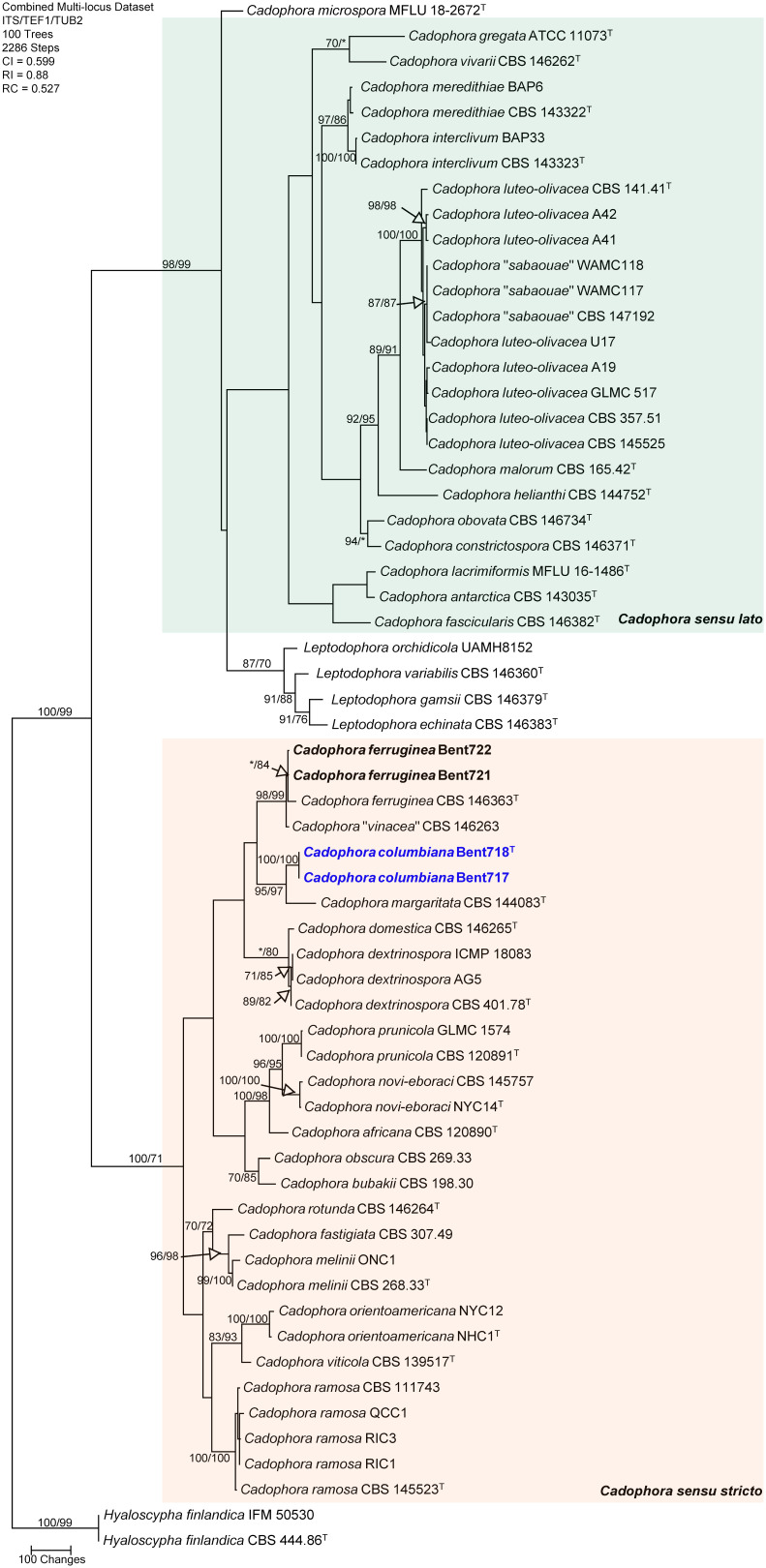
Phylogram generated from maximum parsimony analysis of the genus *Cadophora* based on a combined ITS, TEF1-α and TUB sequence dataset. Bootstrap support values for MP and ML equal to or greater than 70% are presented. The tree is rooted to *Hyaloscypha finlandica* (CBS 444.86 and IFM 50530). The new isolates are in bold and isolates of new species are in blue. Ex-type strains are noted with superscript T.

The concatenated sequences (ITS, TUB and TEF-1α) of the four *Cadophora* isolates recovered in this study along with those of 57 representative isolates (including two isolates of the outgroup species *Hyaloscypha finlandica*; **Supplementary Table 2**) were subjected to ML and MP analyses. For ML analyses, the best-fit model of nucleotide substitution was deduced based on the AIC criterion (ITS: K2+G+I; TEF-1α: HKY+G; TUB: HKY+G+I). The concatenated sequence alignment resulted in a 1,689 character dataset (834 characters were constant, 682 characters were parsimony-informative, and 173 characters were parsimony-uninformative). MP analysis produced 100 equally most parsimonious trees of 2,286 steps and a consistency index (CI), retention index (RI), and rescaled consistency index (RC) of 0.599, 0.88 and 0.527, respectively. The phylogeny consisted of two main clades supported by MP and ML analyses (98/99% and 100/71% MP and ML bootstrap values, respectively). Within the first main clade (98/99% support), including *Cadophora sensu lato* following Koukol and Maciá-Vicente (2022), all known species were well-supported as independent phylogenetic lineages, except that three isolates of the recently described *Cadophora sabaouae* species did cluster with strong support (100/100%) with eight isolates of the well-known species *Cadophora luteo-olivacea*, and these three isolates were placed on a branch internal to the one of the type specimen for this species (CBS 141.41). Within the second main clade (*Cadophora sensu stricto*) that includes the type species for the genus (*Cadophora fastigiata*), MP and ML analyses of the dataset revealed that two isolates (Bent721 and Bent722) clustered strongly (98/99%) with the type specimen of *Cadophora ferruginea* (CBS 146363) but also with the type specimen (CBS 146263) associated with the recently described species *Cadophora vinaceae* (**Figure 1**). The isolate of *Cadophora vinaceae* CBS 146263 was placed on a short branch within this clade, indicating very short phylogenetic distance between this isolate and the type specimen of *Cadophora ferruginea*. Two isolates (Bent717 and Bent718) formed a well-supported clade (100/100%) that does not include any previously described species. This clade was strongly supported as sister clade (95/97%) to the type specimen of *Cadophora margaritata* (CBS 144083), this latter isolate being placed on a moderately long branch in this clade grouping the two species hence supporting important phylogenetic distance between them.

### Phylogenetic analyses of the genus *Cytospora*

**Figure 2:**
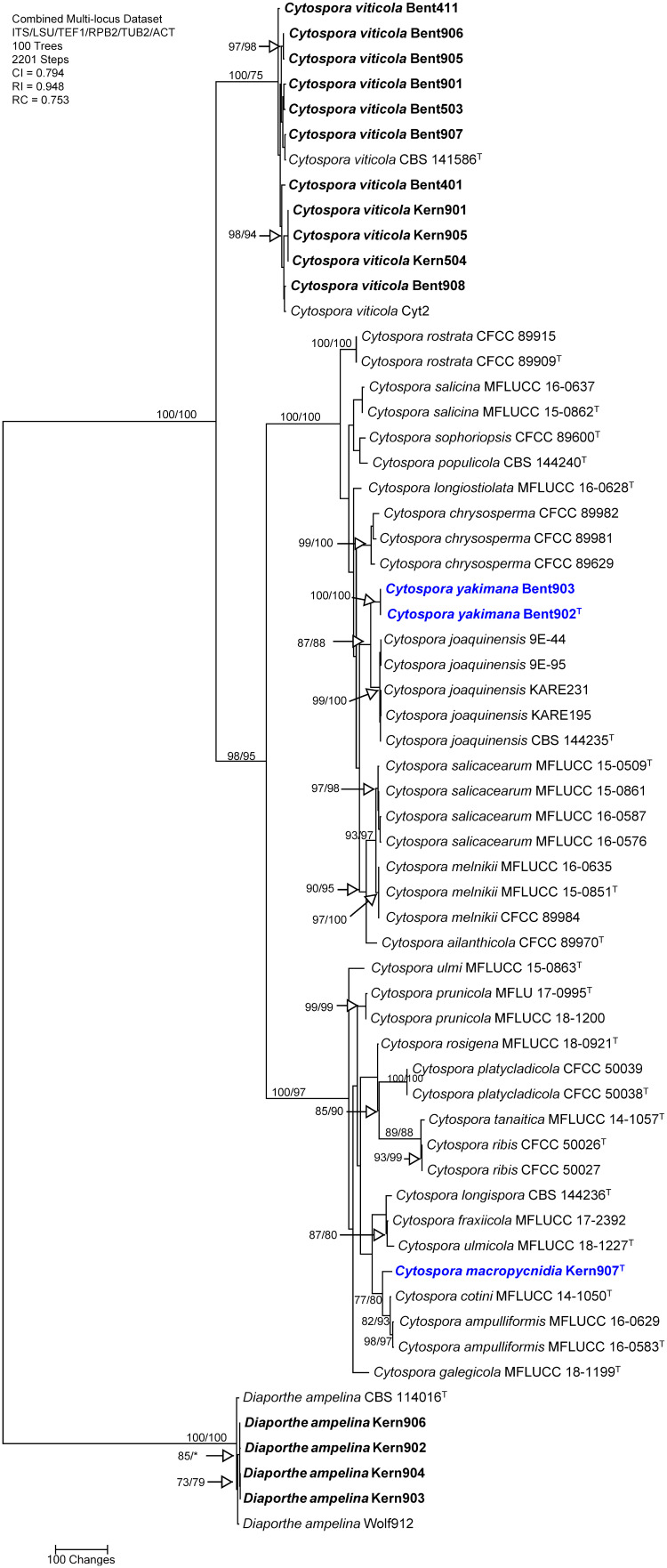
Phylogram generated from maximum parsimony analysis of the genus *Cytospora* based on a combined ITS, LSU, TEF-1α, RPB2, TUB and ACT sequence dataset. Bootstrap support values for MP and ML equal to or greater than 70% are presented. The tree is rooted to *Diaporthe ampelina* (CBS 114016 and Wolf912). The new isolates are in bold and isolates of new species are in blue. Ex-type strains are noted with superscript T. .

The concatenated sequences (ITS, LSU, TEF-1α, RPB2, TUB and ACT) of the 14 *Cytospora* isolates recovered in this study, along with those of 43 representative isolates (including two isolates of the outgroup species *Diaporthe ampelina*; **Supplementary Table 3**), and four isolates of *Diaporthe ampelina* from this study were subjected to ML and MP analyses. For ML analyses, the best-fit model of nucleotide substitution was deduced based on the AIC criterion (ITS: GTR+G+I; LSU: TrN+G+I; TEF-1α: K2+G+I; RPB2: TrN+G+I; TUB: HKY+G; ACT: K2+G+I). The concatenated sequence alignment resulted in a 4,510 character dataset (3,130 characters were constant, 1,100 characters were parsimony-informative, and 280 characters were parsimony-uninformative). MP analysis produced 100 equally most parsimonious trees of 2,201 steps and a CI, RI, and RC of 0.794, 0.948 and 0.753, respectively. One early-divergent, well-supported clade (100/75%) included 11 isolates from the current study and was identified as *Cytospora viticola*, based on their clustering with the type specimen for this species (CBS 141586). Two other well-supported, main clades (100/100% and 100/97% respectively) were revealed by MP and ML analyses (**Figure 2**), with one clade including the type species for the genus, *Cytospora chrysosperma*, and one clade including species such as *Cytospora ribis* and *Cytospora cotini*. Within the *Cytospora chrysosperma* clade, MP and ML analyses revealed that two isolates (Bent902 and Bent903) formed a well-supported clade (100/100%) that does not include any previously described species. This clade was supported as sister clade (87/88%) to the one including the type specimen for *Cytospora joaquinensis* (CBS 144235). Within the *Cytospora ribis* main clade, MP and ML analyses revealed that one isolate (Kern907) represented a unique phylogenetic lineage moderately supported as sister taxon (77/80%) to *Cytospora cotini* and *Cytospora ampulliformis*.

### Phylogenetic analyses of the genus *Sporocadus*

**Figure 3:**
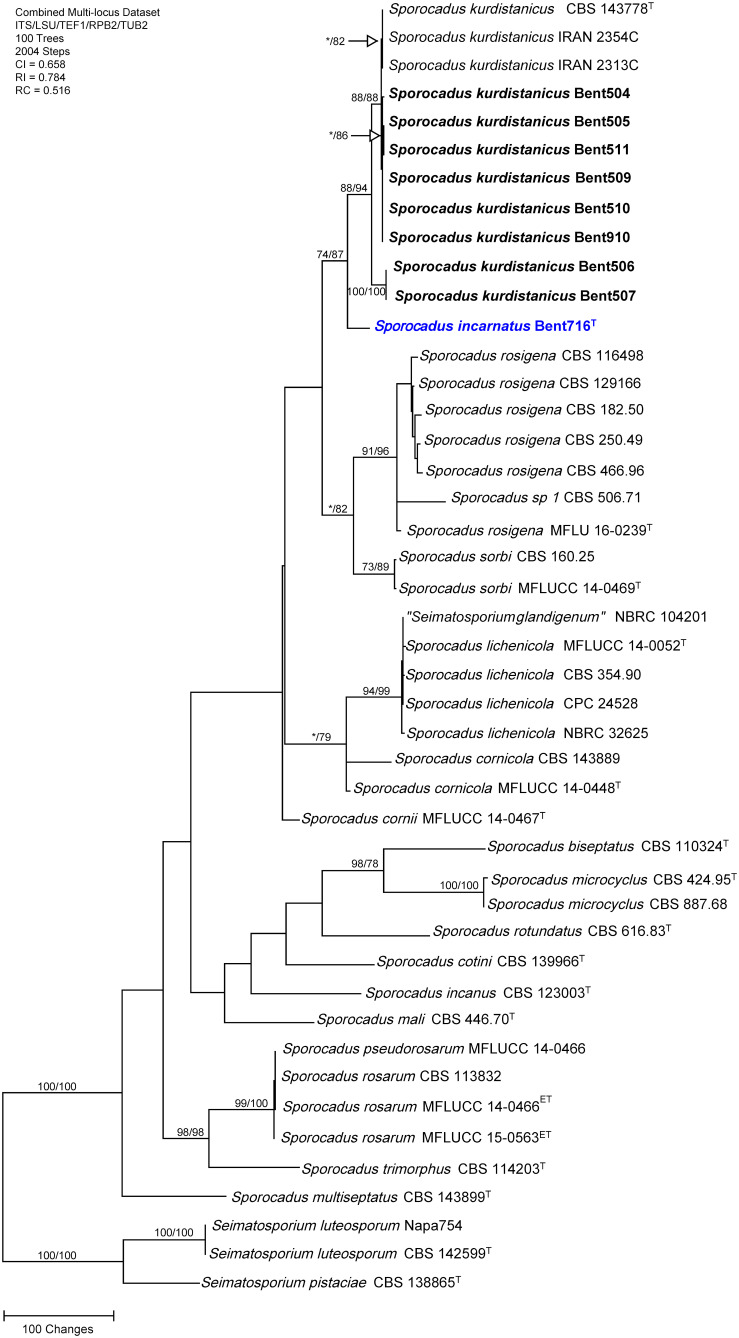
Phylogram generated from maximum parsimony analysis of the genus *Sporocadus* based on a combined ITS, LSU, TEF-1α, RPB2, and TUB sequence dataset. Bootstrap support values for MP and ML equal to or greater than 70% are presented. The tree is rooted to *Seimatosporium luteosporum* (CBS 142599 and Napa754) and *Seimatosporium pistaciae* (CBS 138865). The new isolates are in bold and isolates of new species are in blue. Ex-type strains are noted with superscript T.

The concatenated sequences (ITS, LSU, TEF-1α, RPB2, and TUB) of the nine *Sporocadus* isolates recovered in this study, along with those of 36 representative isolates (including two isolates of the outgroup species *Seimatosporium luteosporum*, and one isolate of the outgroup species *Seimatosporium pistaciae*; **Supplementary Table 4**) were subjected to ML and MP analyses. For ML analyses, the best-fit model of nucleotide substitution was deduced based on the AIC criterion (ITS: T92+G; LSU: K2+G+I; TEF-1α: HKY+G+I; RPB2: T92+G; TUB: HKY+G+I). The concatenated sequence alignment resulted in a 3,908 character dataset (2,940 characters were constant, 639 characters were parsimony-informative, and 329 characters were parsimony-uninformative). MP analysis produced 100 equally most parsimonious trees of 2,004 steps and a CI, RI, RC of 0.658, 0.784 and 0.516, respectively. Eight isolates were placed in a well-supported clade (88/94%), including the type specimen for the recently described species *Sporocadus kurdistanicus* (CBS 143778). The ninth isolate (Bent716) was placed on a relatively long branch outside this clade (74/87% support) and did not cluster with a type or non-type isolate with DNA sequence data available (**Figure 3**); isolate Bent716 represented a novel phylogenetic species.

### Taxonomy

Based on DNA sequence data and morphological examinations, two isolates represent an undescribed *Cadophora* species, three isolates represent two undescribed *Cytospora* species, and one isolate represents an undescribed *Sporocadus* species. These newly discovered species are described below.

*Cadophora columbiana* Travadon & D.P. Lawr., sp. nov.

MycoBank No: MB844778

**Figure 4**

**Figure 4:**
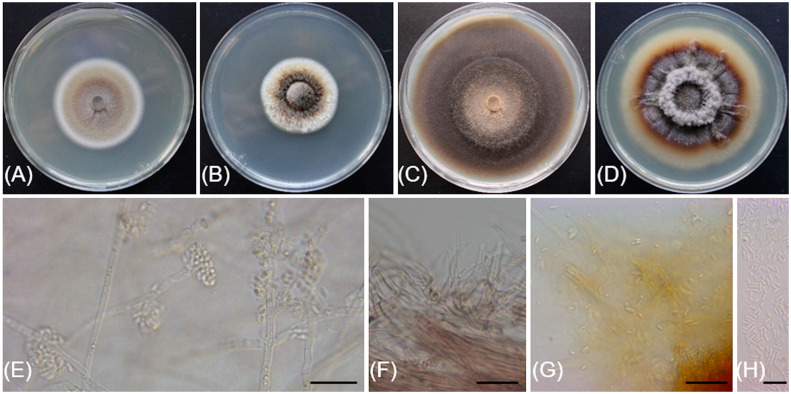
*Cadophora columbiana* (CBS 149299/Bent718). **(A, B)** 14-day-old colonies on MEA and PDA media, respectively. **(C, D).** 55-day-old colonies on MEA and PDA media, respectively. **(E)**. Conidiophores, conidiogenous cells and conidia aggregated in heads. **(F)** Phialides and colarettes. **(G, H).** Conidia. Scale bars: 20 µm **(E–G)**; 10 µm **(H)**.

Typification: USA, Washington: Skamania County, 45°44'13.05"N, 121°34'32.40"W, 557m asl, isolated from necrotic wood of *Vitis vinifera* ‘Pinot noir’, August 2018, R. Travadon number Bent718 (holotype BPI 911228, dried culture; ex-type CBS 149299). ITS sequence GenBank OP038027; TEF-1α sequence GenBank OP106917; TUB sequence GenBank OP079860.

Etymology: The epithet refers to the location from which the fungus originates, in a vineyard near the Columbia River in Washington State, USA.

Description: *Sexual morph* not observed. *Asexual morph* observed in culture on 2% malt extract agar (MEA). *Vegetative mycelium* hyaline, smooth-walled, septate, branched, 1.4-(2.3)-3.2 µm wide, becoming brown with age, chlamydospores not observed. *Conidiophores* arising from aerial hyphae, hyaline, smooth-walled, septate, short, 5.2-(11.6)-23.2 µm long. *Conidiogenous cells* hyaline, smooth-walled, terminal or lateral, monophialidic, obclavate, with a flask-shaped appearance, 6.7-(12.7)-20.1 µm long × 2.7-(3.5)-4.2 µm wide at the widest part, tapering towards collarette; collarettes flaring, cup-shaped, 1.4-(1.8)-2.3 µm wide at upper edge. *Conidia* hyaline, smooth-walled, aseptate, ovoid to ellipsoidal, enteroblastic, aggregated in heads, 2.9-(4.8)-6.7 × 1.2-(1.8)-2.5 µm.

*Culture characteristics*: Colonies on MEA reaching 28 mm diam after 13 d at 20°C in the dark. Flat colony with undulate margin, color ranging from isabelline to honey to off-white from center to edge. Colonies on 3.9% potato dextrose agar (PDA) displaying aerial, fasciculate hyphae with aerial mycelium tufts at the center, margin undulate, colony color ranging from isabelline to dark isabelline to off-white from center to edge.

Notes: Based on the phylogenetic inference obtained in this study, *Cadophora columbiana* is the closest relative to *Cadophora margaritata*, with 95% Maximum Parsimony bootstrap support. Both species are lignicolous and characterized by phialidic conidiogenesis with non-septate conidia attached in heads. Colony colors on MEA are very distinct between the two species, with *Cadophora margaritata* being olivaceous black and *Cadophora columbiana* ranging from isabelline to white. *Cadophora margaritata* CBS 144083 produces longer phialides (up to 29 µm) than *Cadophora columbiana* Bent718 (up to 13.6 µm), and conidia of the former species are truncated at the base, a characteristic not observed for those of *Cadophora columbiana*.

The ITS sequence of *Cadophora columbiana* Bent718 differs at four nucleotide positions (99% identity) from that of the ex-holotype of *Cadophora margaritata* CBS 144083, whereas the TUB sequences differ at 72 nucleotide positions (84% identity).

Additional specimens examined: USA, Washington: Benton County, 45°44'13.05"N, 121°34'32.40"W, 557 m asl, isolated from necrotic wood of *Vitis vinifera* ‘Pinot noir’, August 2018, R. Travadon number Bent717 (CBS 149300).

*Cytospora macropycnidia* Travadon & D.P. Lawr., sp. nov.

MycoBank No: MB844777

**Figure 5**

**Figure 5:**
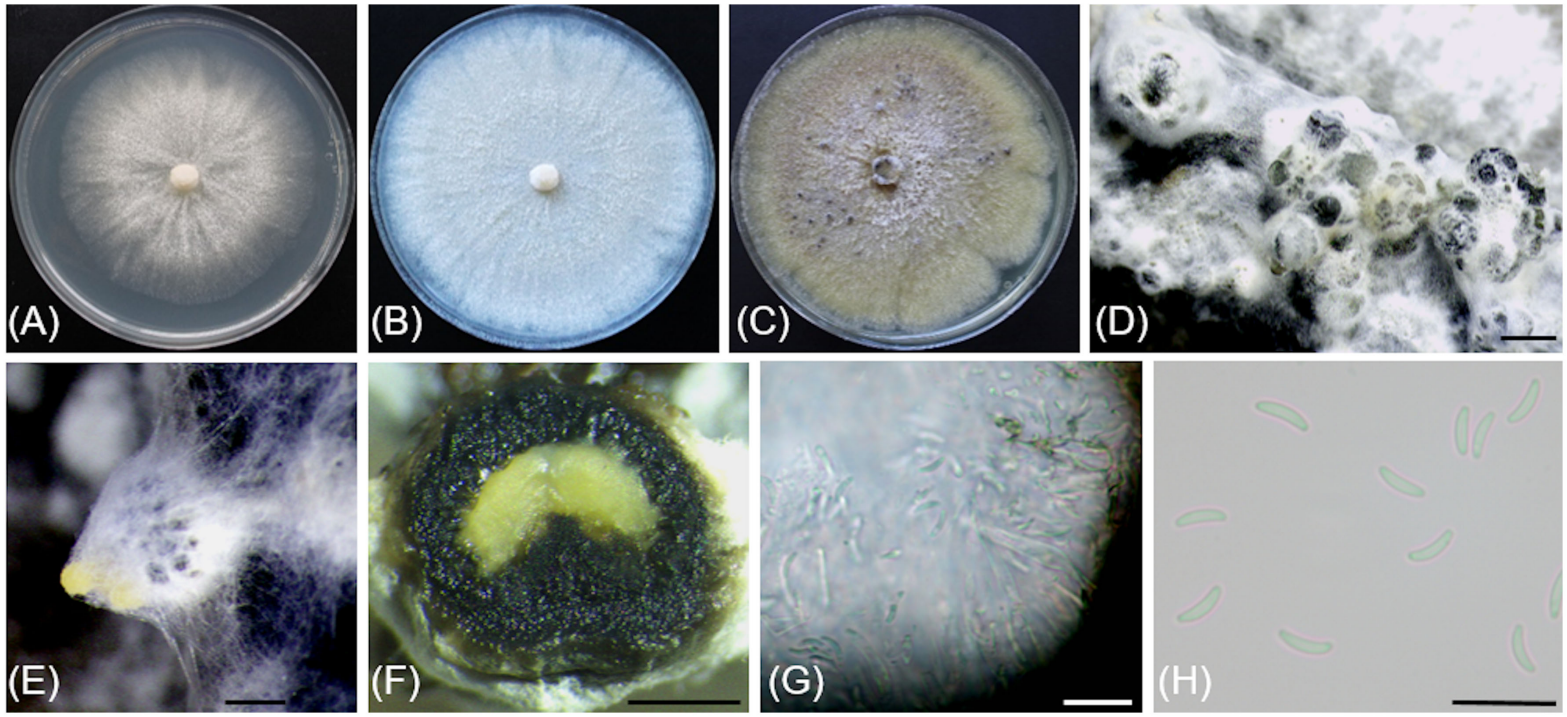
*Cytospora macropycnidia* (CBS 149338/Kern907). **(A)** 4-day-old culture on 2% PDA. **(B)** 7**-**day-old culture on 2% PDA. **(C)** 32-day-old culture on 3.5% PDA. **(D)** Gregarious pycnidia produced *in vitro* on sterile grapevine wood. **(E)** Solitary pycnidium exudating pale yellow cirrhus (mucilage). **(F)** Longitudinal section of a pycnidium. **(G)** Conidiogenous cells and conidia. **(H)** Conidia. Scale bars: 1mm **(D–F)**; 10µm **(G, H)**.

Typification: USA, California: Kern County, 35°42'13.8"N, 119°23'16.1"W, 77m asl, isolated from necrotic wood of *Vitis vinifera* ‘Scarlet Royal’, October 2018, R. Travadon number Kern907 (holotype BPI 911229; dried culture; ex-type CBS 149338). ITS sequence GenBank OP038094; LSU sequence GenBank OP076935; ACT sequence GenBank OP003977; RPB2 sequence GenBank OP095265; TEF-1α sequence GenBank OP106954; TUB sequence GenBank OP079909.

Etymology: The epithet refers to the large pycnidia, which form on grapevine wood in the laboratory.

Description: *Sexual morph* not observed. *Asexual morph* observed in culture on 2% potato dextrose agar (PDA). *Conidiomata* pycnidial, ostiolated, black to mouse grey with white to off-white surface hyphae, 2.3-(3.2)-4.3 mm diameter, erumpent on bark surface, solitary and gregarious, with multiple internal locules sharing invaginated walls, conceptacle absent. *Mycelium* hyaline, smooth-walled, septate, branched, 2.8-(4.4)-5.6 µm wide. *Conidiophores* reduced to conidiogenous cells. *Conidiogenous cells* hyaline, smooth-walled, with a flask-shaped appearance, 13.3-(17)-23.6 µm long × 2-(2.5)-2.9 µm wide. *Conidia* hyaline, allantoid, smooth, aseptate, 4.9-(6)-7.6 µm long × 1.7-(2.1)-2.4 µm wide.

*Culture characteristics*: Colonies on PDA reaching 85 mm diam after 5 d at 25°C in the dark, fast-growing with relatively even but slightly dentate margin, white to off-white with short aerial tufts giving a cottony appearance, becoming straw-colored with age.

Notes: Based on the phylogenetic inference obtained in this study, *Cytospora macropycnidia* forms a moderately supported clade (77% Maximum Parsimony and 80% Maximum Likelihood bootstrap support) with *Cytospora ampulliformis* (MFLUCC 16-0583 isolated from *Sorbus intermedia* in Russia) and *Cytospora cotini* (MFLUC 14-1050 isolated from *Cotinus coggygria* in Russia). *Cytospora macropycnidia* has larger pycnidia (average 3.2 mm in diameter) than *Cytospora ampulliformis* and *Cytospora cotini* (< 1 mm).

The ITS sequence of *Cytospora macropycnidia* Kern907 differs at three nucleotide positions (99% identity) from those of both *Cytospora ampulliformis* MFLUCC 16-0583 and of *Cytospora cotini* MFLUC 14-1050. The LSU sequence of *Cytospora macropycnidia* Kern907 differs at three nucleotide positions (99% identity) from that of *Cytospora ampulliformis* MFLUCC 16-0583 and has 100% identity with that of *Cytospora cotini* MFLUC 14-1050. The RPB2 sequence of *Cytospora macropycnidia* Kern907 differs at 12 nucleotide positions (98% identity) from that of *Cytospora ampulliformis* MFLUCC 16-0583 and at 22 nucleotide positions (98% identity) from that of *Cytospora cotini* MFLUC 14-1050. The ACT sequence of *Cytospora macropycnidia* Kern907 differs at six nucleotide position (98% identity) from that of *Cytospora ampulliformis* MFLUCC 16-0583.

*Cytospora yakimana* Travadon & D.P. Lawr., sp. nov.

MycoBank No: MB844775

**Figure 6**

**Figure 6:**
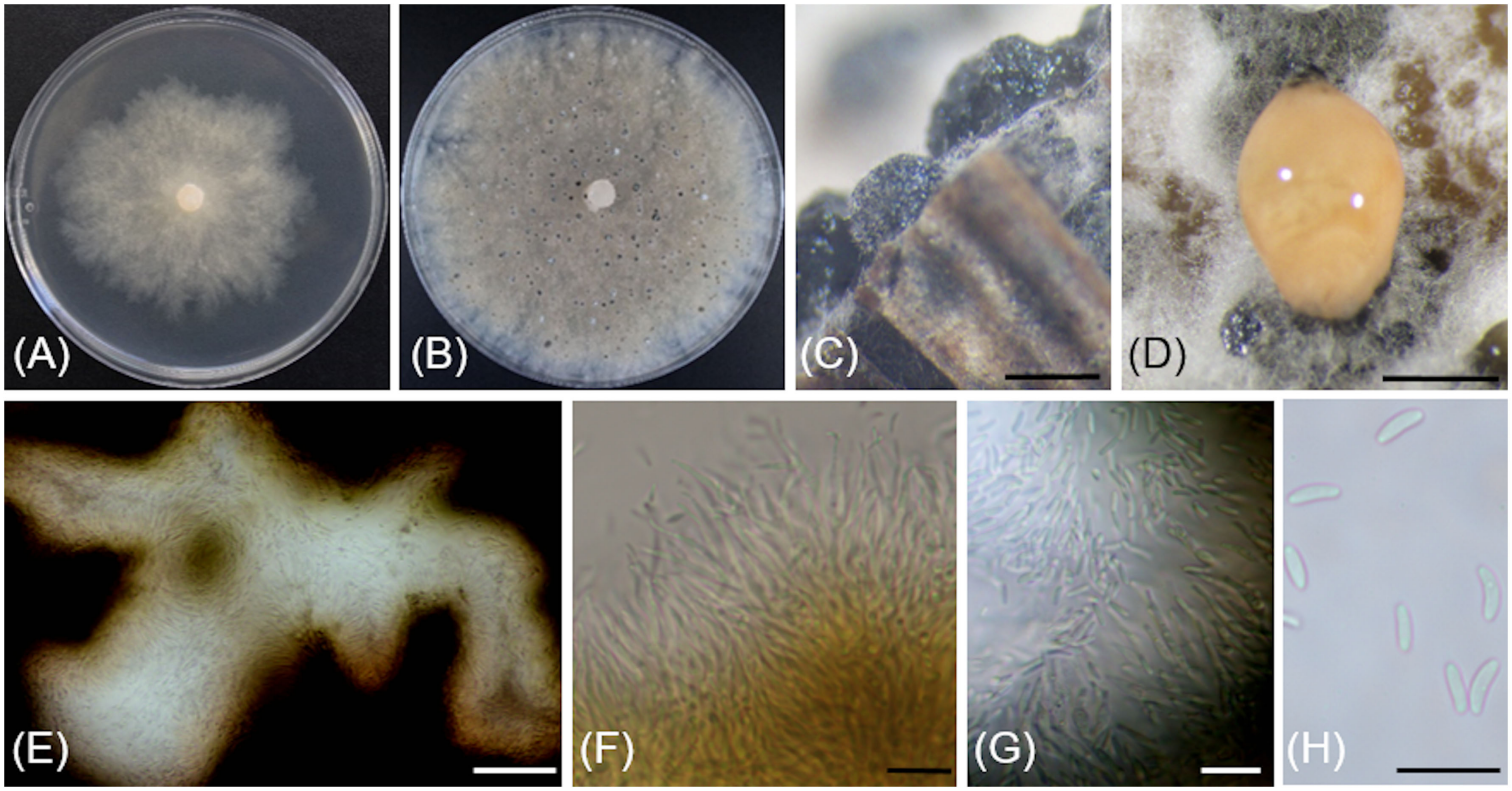
*Cytospora yakimana* (CBS 149297/Bent902). **(A)** 4-day-old culture on PDA. **(B)** 14**-**day-old culture on PDA. **(C)** Pycnidia produced *in vitro* on sterile grapevine wood. **(D)** Pycnidium exudating pale luteous to saffron colored mucilage. **(E)** Locular chamber lined with conidiogenous cells. **(F)** Straight and branched conidiogenous cells. **(G)** Conidiogenous cells and conidia. **(H)** Conidia. Scale bars: 1 mm **(C, D)**; 50 µm **(E)**; 10 µm **(F–H)**.

Typification: USA, Washington: Benton County, 46°12'53.7"N, 119°44'47.9"W, 208m asl, isolated from necrotic wood of *Vitis vinifera* ‘Tinto Cão’, October 2018, R. Travadon number Bent902 (holotype BPI 911230; dried culture; ex-type CBS 149297). ITS sequence GenBank OM976602; LSU sequence GenBank ON059350; ACT sequence GenBank ON012555; RPB2 sequence GenBank ON045093; TEF-1α sequence GenBank ON012569; TUB sequence GenBank ON086750.

Etymology: The epithet refers to the Yakima River in Washington State, USA, bordering the vineyard where the specimen was originally isolated.

Description: *Sexual morph* not observed. *Asexual morph* observed in culture on sterile grapevine wood embedded in 3.9% potato dextrose agar (PDA). *Conidiomata* pycnidial, ostiolated, mouse grey with white to off-white surface hyphae, 1.1-(2)-2.4 mm diameter, erumpent or semi-immersed on bark surface, mostly solitary, rarely aggregated, without conceptacle, with multiple internal locules with shared invaginated walls. *Mycelium* hyaline, smooth-walled, septate, branched, 2.3-(3.9)-5 µm wide. *Conidiophores* hyaline, smooth-walled, reduced to 2—3 monoblastic, branching filamentous conidiogenous cells. *Conidiogenous cells* hyaline, smooth-walled, tapering towards the apex, 11-(14.9)-18.6 µm long × 0.7-(0.9)-1.4 µm wide. *Conidia* hyaline, allantoid, smooth, aseptate, 3.9-(4.3)-4.9 µm long × 1.4-(1.8)-2 µm wide.

*Culture characteristics*: Colonies on 2% PDA reaching 70 mm diam after 7 d at 25°C in the dark, moderately fast-growing with an uneven margin, white to off-white with hints of pale rose with age.

Notes: Based on the phylogenetic inference obtained in this study, *Cytospora yakimana* is the closest relative to *Cytospora joaquinensis*, with 87% Maximum Parsimony and 88% Maximum Likelihood bootstrap support. *Cytospora yakimana* originates from *Vitis vinifera* in Washington state, whereas *Cytospora joaquinensis* is reported from *Juglans regia*, *Pistacia vera* and *Populus deltoides* in California. *Cytospora yakimana* produces conidiogenous cells that may be branched, which is not a culture characteristic of *Cytospora joaquinensis*. Conidia of *Cytospora yakimana* (3.9 to 4.9 µm long) are slightly shorter than those of *Cytospora joaquinensis* (5 to 6 µm long).

The ITS and ACT sequences of *Cytospora yakimana* Bent902 both differ at only one nucleotide position (99% identity) from those of the ex-holotype of *Cytospora joaquinensis* CBS 144235. However, the TEF-1α sequence of *Cytospora yakimana* Bent902 differs at 22 nucleotide positions (92% identity) from that of *Cytospora joaquinensis* CBS 144235, whereas the TUB sequences of these two isolates differ at 17 nucleotide positions (96% identity).

Additional specimens examined: USA, Washington: Benton County, 46°12'53.7"N, 119°44'47.9"W, 208m asl, isolated from necrotic wood of *Vitis vinifera* ‘Tinto Cão’, October 2018, R. Travadon number Bent903 (CBS 149298).

*Sporocadus incarnatus* Travadon & D.P. Lawr., sp. nov.

MycoBank No: MB844774

**Figure 7**

**Figure 7:**
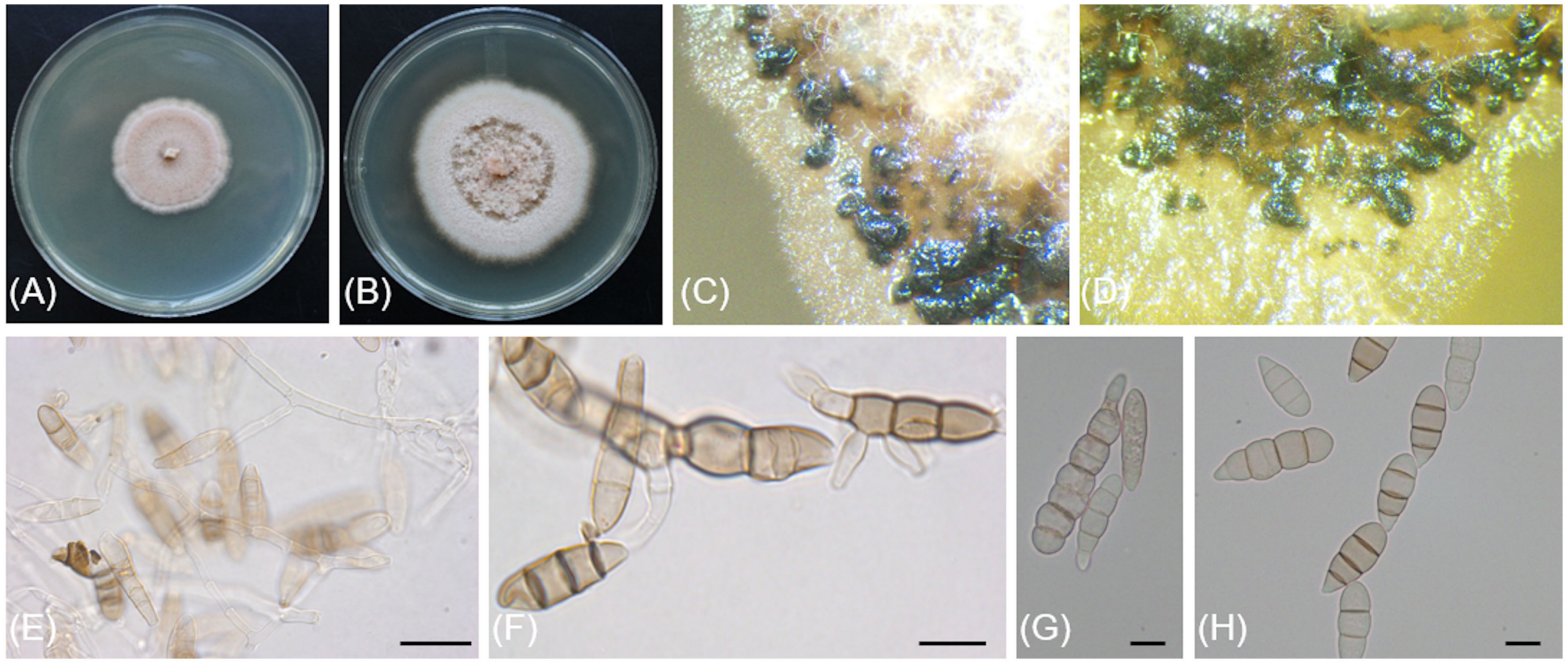
*Sporocadus incarnatus* (CBS 149301/Bent716). **(A, B)** 14-day-old colonies on MEA and PDA media, respectively. **(C, D)**. Conidiomata on PDA. **(E)** Conidiophores, conidiogenous cells and conidia. **(F)** Germinating conidia. **(G, H)** Conidia. Scale bars: 20µm **(E)**; 10µm **(F–H)**.

Typification: USA, Washington: Benton County, 46°17'56.8"N, 119°46'53.1"W, 370m asl, isolated from necrotic wood of *Vitis vinifera* ‘Riesling’, August 2018, R. Travadon number Bent716 (holotype BPI 911231; dried culture; ex-type CBS 149301). ITS sequence GenBank OP038025; LSU sequence GenBank OP076913; RPB2 sequence GenBank OP095241; TUB sequence GenBank OP079858.

Etymology: The epithet refers to the flesh to salmon color of the colony when grown in culture.

Description: *Sexual morph* not observed. *Asexual morph* observed in culture on 3.9% potato dextrose agar (PDA). *Conidiomata* black, erumpent, semi-immersed or immersed, mainly gregarious, sometimes scattered. *Mycelium* hyaline, smooth-walled, septate, branched, 2.9-(3.9)-4.8 µm wide. *Conidiophores* hyaline, smooth-walled, septate, up to 60 µm. *Conidiogenous cells* hyaline, smooth-walled, mainly cylindrical but occasionally ampulliform, 8.6-(17.7)-28.9 µm long × 2.5-(3.4)-4.2 µm wide. *Conidia* ovoid to subcylindrical, straight, 2—7-septate, wall smooth, often slightly constricted at the septa, 20-(28)-46.5 × 8.2-(9.2)-10.8 µm, lacking appendages; basal cell obconic, occasionally with a truncate base, hyaline to pale brown, thin-walled, 3.6-(6.3)-9.7 µm long; median cells 2—5 with fairly thick walls, pale to mid-brown, dolioform to subcylindrical, 4-(6)-8.7 µm long; apical cell obtuse, concolorous with median cells, 5.3-(6.6)-9.2 µm long.

*Culture characteristics*: Colonies on PDA reaching 63 mm diam after 14 d at 25°C in the dark. Colony displaying aerial mycelium tufts at the center, margin irregular, colony color ranging from cinnamon to fleshish salmon to off-white from center to edge. Colonies on 2% malt extract agar (MEA) flat with irregular margin, color ranging from saffron to fleshish salmon to saffron to off-white from center to edge.

Notes: Based on the phylogenetic inference obtained in this study, *Sporocadus incarnatus* is the closest relative to *Sporocadus kurdistanicus*, with 74% Maximum Parsimony and 87% Maximum Likelihood bootstrap support. Both species have been isolated from necrotic wood of *Vitis vinifera*. Colony colors on PDA are distinct between the two species, with *Sporocadus kurdistanicus* described as buff to sepia whereas *Sporocadus incarnatus* color ranges from flesh to salmon. Growth rate for *Sporocadus kurdistanicus* is higher (48 to 56 mm after 14 d at 20°C, compared to 33 to 45 mm after 14 d at 21°C for *Sporocadus incarnatus*). Conidia of *Sporocadus incarnatus* have 2 to 7 septa, whereas those of *Sporocadus kurdistanicus* (like most *Sporocadus* species), have three septa.

The ITS and LSU sequences of *Sporocadus incarnatus* Bent716 both differ at three nucleotide positions (99% identity) from those of the ex-holotype of *S. kurdistanicus* CBS 143778, whereas the TUB sequences differ at 34 nucleotide positions (95% identity).

### Pathogenicity tests

At 12 months after inoculation, the wood surrounding the inoculation sites of water-inoculated control plants was discolored, but the discoloration was restricted (LWD = 6.8 mm; *n* = 53 plants, averaged across experiments) and no pathogenic fungi were isolated from these lesions (**Table 2**). The woody stems of plants inoculated with *Phaeomoniella chlamydospora* isolate Bent708 (positive-control plants) had black, vascular discolorations spreading above and below the inoculation site, with an average LWD of 19.1 mm (*n* = 62 plants, averaged across experiments). ANOVA revealed a significant effect of inoculation treatment on lesion length (*P*< 0.0001). All inoculated plants had significantly larger lesions than the water-inoculated controls (Dunnett's test; *P*< 0.0001, α = 0.05). These lesions varied in color from light orange to brown to black, depending on the isolate. Plants inoculated with *Flammulina filiformis* Bent008 had significantly smaller lesions (average of 12.7 mm), compared to those inoculated with *Phaeomoniella chlamydospora* Bent708, *Thyrostroma* sp. Bent904, *Biscogniauxia mediterranea* Kern007, *Cadophora columbiana* Bent718, and *Kalmusia variispora* Bent603 (**Table 2**). Plants inoculated with *Cadophora ferruginea*, *Cytospora viticola*, *Cytospora yakimana*, and *Eutypella citricola* had lesions intermediate in length. From the wood lesions of the inoculated plants, we recovered isolates matching morphologically the inoculated isolates, with recovery rates ranging from 43% for *Flammulina filiformis* Bent008 to 67% for *Biscogniauxia mediterranea* Kern007. Both LWD and recovery rates suggest that all ten isolates are indeed pathogenic.

**Table 2:** Mean lesion lengths and recovery rates of ten fungal isolates from the woody stems of *Vitis vinifera* ‘Chardonnay’ at 12 months post-inoculations in duplicated greenhouse experiments.

Inoculation treatment	Lesion length (mm)^1^	95% Confidence limits of lesion length (mm)	Recovery rate (%)^2^	Sample size
Non-inoculated control	6.81a	5.41 - 8.59	0	53
*Flammulina filiformis* Bent008	12.72b	10.18 - 15.90	43.33	30
*Cadophora ferruginea* Bent721	16.95bc	13.56 - 21.19	53.33	30
*Eutypella citricola* Kern004	17.92bc	14.32 - 22.43	57.14	28
*Cytospora viticola* Bent901	18.40bc	14.72 - 22.99	48.48	33
*Cytospora yakimana* Bent902	18.77bc	15.02 - 23.47	60	30
*Phaeomoniella chlamydospora* Bent708	19.12bc	15.02 - 24.35	46.77	62
*Thyrostroma* sp. Bent904	19.75bc	15.80 - 24.68	43.75	32
*Biscogniauxia mediterranea* Kern007	20.38c	16.28 - 25.50	66.67	27
*Cadophora columbiana* Bent718	21.19c	16.92 - 26.54	53.57	28
*Kalmusia variispora* Bent603	21.73c	17.38 - 27.16	46.88	32

^1^ Mean lesion lengths with the same letters have overlapping 95% confidence intervals.

^2^ Recovery rates were estimated as the number of plants from which the isolate was recovered out of the total number of plants inoculated.

### Effect of temperature on mycelial growth

**Figure 8:**
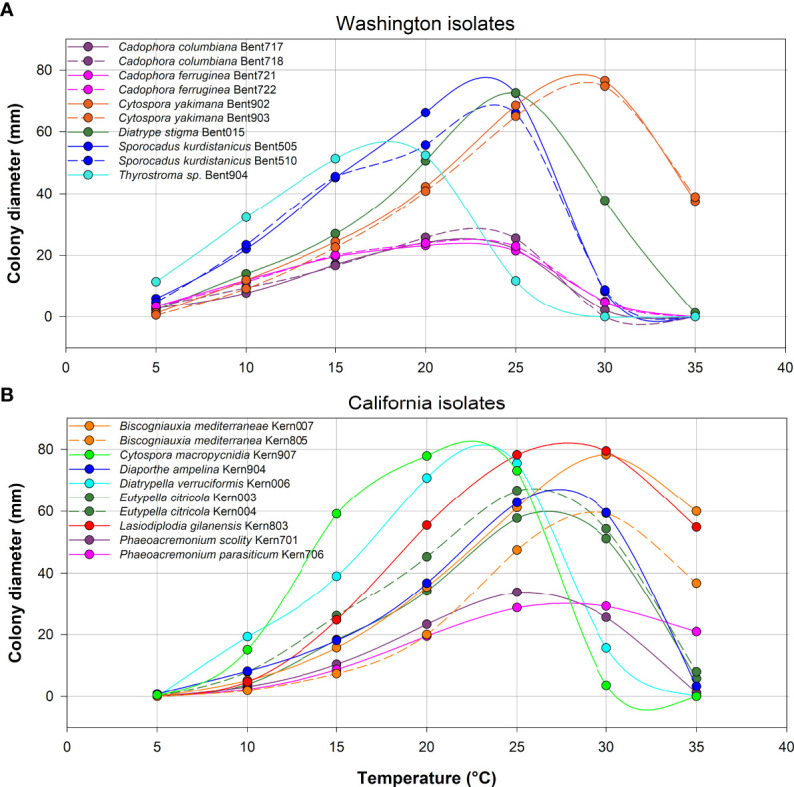
Average colony diameter of ten isolates from Washington **(A)** and ten isolates from California **(B)** assessed at temperatures ranging from 5 to 35°C in 5°C increments. At each temperature, three media dishes were used per isolate in each of two experiments.

All 10 isolates from Washington grew at 5°C, whereas only three of the 10 isolates from California grew at 5°C (*Eutypella citricola* Kern004, *Diaporthe ampelina* Kern904 and *Cytospora macropycnidia* Kern907; **Figure 8**). In contrast, at the highest temperature of 35°C, nine of 10 isolates from California grew (all species except *Cytospora macropycnidia* Kern907), whereas only three of 10 isolates from Washington grew at 35°C (*Diatrype stigma* Bent015, *Cytospora yakimana* Bent902 and Bent903). All isolates from Washington had an optimal growth temperature below 24°C, except for the two *Cytospora yakimana* isolates (optimal temperatures of 27.1 and 27.4°C, respectively; **Supplementary Table 5**). All isolates from California had an optimal growth temperature above 24°C, except for two isolates (*Cytospora macropycnidia* Kern907 at 20.3°C and *Diatrypella verruciformis* Kern006 at 21.5°C; **Supplementary Table 5**). The lowest optimal growth temperature was 16.2°C for *Thyrostroma* sp. Bent904 and the highest optimal growth temperature was 29.8C for *Biscogniauxia mediterranea* Kern004.

## Discussion

We examined the communities of pathogenic fungi associated with esca-like symptomatic vines from two grape production systems and two regions with distinct climates and environmental conditions: southeastern Washington wine grapes and southern San Joaquin Valley of California table grapes. Although our field survey was limited to only 10 vineyards in each region, we established a fungal collection of more than 110 isolates representing 36 species, either consistently associated with grapevine trunk diseases or known causal agents of dieback in other woody plants. For the first time, we report esca in vineyards from Washington, which was expected, as this disease is present in virtually all grape-growing regions of western North America, including neighboring British Columbia (Úrbez-Torres et al., 2014a). A core set of six species was shared between the two regions: *Cytospora viticola*, *Diatrype stigma*, *Diplodia seriata*, *Kalmusia variispora*, *Phaeoacremonium minimum*, and *Phaeomoniella chlamydospora*, These species may thus be adapted to a wide array of environmental and cultural conditions. These species are widely reported as trunk pathogens, except for *Cytospora viticola* and *Kalmusia variispora*, which were more recently characterized as pathogenic in vineyards from the northeastern US and northwestern Iran, respectively (Lawrence et al., 2017a; Abed-Ashtiani et al., 2019).

Members of the genus *Cytospora* include species that cause cankers on a range of horticultural crops, and Cytospora canker is recognized as a common disease in many fruit and nut crops (Adams et al., 2002; Fan et al., 2015; Lawrence et al., 2018a). However, *Cytospora* species are not frequently reported causal fungi of grapevine trunk diseases [see Fotouhifar et al. (2010); Lawrence et al. (2017a) and Dekrey et al. (2022). In Washington vineyards, *Cytospora viticola* was found at two sites on four distinct cultivars, and in California, it was found at three sites and three cultivars. This is a first report of *Cytospora viticola* in these two states; this species was previously reported causing Cytospora canker in vineyards from the northeastern US (US states of Connecticut, Michigan, Minnesota, New York, Ohio, Vermont, Virginia, and Wisconsin), and southeastern Canada (Canadian provinces of Ontario and Québec) (Lawrence et al., 2017a; Dekrey et al., 2022). Our results of controlled inoculations confirmed *Cytospora viticola* as pathogenic to grapevine wood and support Cytospora canker as a grapevine trunk disease. Like Botryosphaeria and Phomospsis dieback of grapevines, Cytospora canker is not associated with peculiar foliar symptoms, but instead causes reduced vine vigor and the gradual death of spurs and cordons. It appears that several species of *Cytospora* can be considered pathogenic to grapevine, including *Cytospora vinaceae* (Lawrence et al., 2017a), but also the presently described species *Cytospora yakimana*, which was as virulent as *Cytospora viticola*. In addition, we discovered and described a new species from table grape ‘Scarlet Royal’ in California, *Cytospora macropycnidia*, but further studies are required to evaluate its pathogenicity. *Cytospora* species are among the most prevalent species reported in some grape-growing regions (Dekrey et al., 2022), multiple *Cytospora* species have been shown to naturally infect grapevine pruning wounds in Washington (Baumgartner et al., 2022), and many *Cytospora* species are damaging pathogens of woody hosts (Spielman, 1985; Lawrence et al., 2018a; Pan et al., 2020). The contribution of *Cytospora* species to grapevine dieback may have been overlooked in the past.

Species from the Diatrypaceae family were well represented in our survey, with five species from California table grapes (*Cryptovalsa ampelina*, *Diatrype stigma*, *Diatrypella verruciformis*, *Eutypella citricola* and *Eutypella microtheca*) and six species from Washington wine grapes (*Diatrype stigma*, *Diatrype whitmanensis*, *Eutypa cerasi*, *Eutypa lata*, *Eutypa petrakii* var *hederae*, and *Eutypella virescens*). All five California species are reported there (Trouillas et al., 2010) and most species have been proven vascular pathogens of grapevines, except *Diatrypella verruciformis*, which is considered saprophytic (Trouillas and Gubler, 2010). Inoculation assays confirmed *Eutypella citricola* pathogenic to grapevine and these findings support the role of this species as a trunk pathogen, as previously demonstrated for this species and *Eutypella microtheca* (Trouillas et al., 2011; Luque et al., 2012; Úrbez-Torres et al., 2020). *Eutypella citricola* is best known as a canker pathogen of *Citrus* sp. (Mayorquin et al., 2016). Diatrypaceae species frequently infect a large diversity of cultivated and wild, perennial hosts in the same landscape (Moyo et al., 2019), and have sometimes been shown to be able to cross-infect these distinct hosts (Travadon and Baumgartner, 2015). Because the southern San Joaquin Valley includes both vineyards and citrus orchards, it is possible there are infection routes between these crops. The most common Diatrypaceae species in Washington wine grapes was *Eutypa lata*, the only species that has been proven responsible for causing Eutypa dieback foliar symptoms. Such foliar symptoms were observed during the present survey and previously from another study in the same region (Johnson and Lunden, 1987), hence the presence of *Eutypa lata* is confirmed (Glawe et al., 1982). A comprehensive taxonomic study of Diatrypaceous fungi from Washington, Oregon and Idaho described 28 species, collected from dead or declining angiosperms, with all species belonging to the genera *Cryptosphaeria*, *Cryptovalsa*, *Diatrype*, *Diatrypella*, *Eutypa*, and *Eutypella*, with only *Eutypa lata* associated with *Vitis vinifera* and *V. labruscana* (Glawe and Rogers, 1984). Among the less common species found in Washington, our study constitutes the first report of *Eutypa cerasi* on *V. vinifera*; this is a first report of *Eutypa cerasi* in North America, as it was only known from *Prunus cerasus* in China (Long et al., 2021). We also report for the first time *Eutypa petrakii* var *hederae* on *Vitis vinifera*, as this species was only known from *Hedera helix* in France and Switzerland (Rappaz, 1987). The pathogenicity to grapevine of these two latter *Eutypa* species requires further evaluation.

Multiple isolates of the genus *Sporocadus* were isolated from four sites in Washington and they represented the third most abundant group of fungi in this region. Members of the family Sporocadaceae (order Xylariales) are ceolomycetous fungi also referred to as ‘pestalotioid fungi’ as they produce multiseptate conidia that often bear apical and/or basal appendages. Among the 30 genera in this family (Liu et al., 2019), some have been characterized as pathogenic to grapevine, such as *Seimatosporium* (Díaz et al., 2012; Lawrence et al., 2018b), *Truncatella*, *Pestalotiopsis* and *Neopestalotiospis* (Úrbez-Torres et al., 2009; Arzanlou et al., 2013; Jayawardena et al., 2015; Maharachchikumbura et al., 2017). More recently, three newly described species of pestalotioid fungi were isolated from diseased vines from the Kurdistan Province of Iran, *Seimatosporium marivanicum*, *Sporocadus kurdistanicus*, and *Xenoseimatosporium kurdistanicum*; however, their pathogenicity could not be demonstrated in short-term field pathogenicity trials and they were thus considered saprophytic (Moghadam et al., 2022). Phylogenetic analyses of a dataset combining sequences of five loci grouped eight Washington isolates with the type specimen of *Sporocadus kurdistanicus*, which is here reported for the first time in North America. Two Washington isolates (Bent506 and Bent507) formed a sister clade to this species, but morphological observations supported their affiliation with *Sporocadus kurdistanicus* (data not shown). In contrast, Washington isolate Bent716 was strongly supported as a new species, sister to *Sporocadus kurdistanicus*, but placed on a long branch outside that clade in the phylogeny. Moreover, morphological observations confirmed this distinction with isolate Bent716 producing conidia with up to seven septa, whereas those of *Sporocadus kurdistanicus* have three septa, like most *Sporocadus* species (Liu et al., 2019). The species *Sporocadus incarnatus* is newly described and typified to accommodate isolate Bent716, which was deposited in two public repositories for future investigations of its ecology. Future research should determine the pathogenic status of isolates from this genus in long-term experiments, as they are reported from diseased grapevines around the world (Mundy et al., 2020; Moghadam et al., 2022).

Since our surveys principally targeted vineyards with a history of esca symptoms, it was anticipated to isolate the widely recognized causal agents of this disease, namely *Phaeomoniella chlamydospora* and *Phaeoacremonium minimum*. The frequent white-rot symptoms in the trunks of affected vines are usually associated with species from the phylum Basidiomycota, with *Fomitiporia mediterranea* the main causal agent in European vineyards (Moretti et al., 2021), but multiple basidiomycetes species have been associated with esca worldwide (Fischer, 2006). In California, the predominant species is *Fomitiporia polymorpha* (Brown et al., 2020) and our isolations confirmed the presence of this species in California table grape vineyards. In addition, we recovered two isolates of *Quambalaria cyanescens* (Microstromatales, Quambalariaceae), reporting this species for the first time in North America. This species has recently been categorized as a grapevine trunk pathogen in northwest Iran (Narmani and Arzanlou, 2019) and we hypothesize it may also be part of the complex of fungi causing trunk diseases in California. Basidiomycetes recovered from Washington wine grapes included *Flammulina filiformis* and *Trametes versicolor*, the former species being reported from *Vitis vinifera* for the first time worldwide. As generalist wood-rotting Basidiomycetes, their association with perennial crops such as grapevines is not surprising. *Flammulina filiformis* was the least virulent species in our pathogenicity test. Among the species associated with esca and young vine decline, we identified *Phaeoacremonium parasiticum* and *Phaeoacremonium scolyti* in California, two species that have been isolated from diseased grapevines in various grape-growing regions (Spies et al., 2018). *Phaeoacremonium scolyti* is now reported for the first time in North America.

In addition, two *Cadophora* species were recovered from Washington wine grapes, including the recently described species *Cadophora ferruginea* (Maciá-Vicente et al., 2020), and a new phylogenetic lineage described here as *Cadophora columbiana*. Pathogenicity testing revealed they are both pathogenic to *Vitis vinifera*. In the multilocus phylogenetic analyses of a comprehensive dataset, including all currently known *Cadophora* species, all species were well-supported as independent phylogenetic lineages, except for two taxa: *Cadophora vinaceae* CBS 146263 (Chen et al., 2022), which clustered with strong support with the type isolate of *Cadophora ferruginea* CBS 146363, and *Cadophora sabaouae* (Aigoun-Mouhous et al., 2021), which was nested with strong support with the type isolate of *Cadophora luteo-olivacea* CBS 141.41. *Cadophora vinaceae* CBS 146263 was described as phylogenetically related to *Cadophora ferruginea* CBS 146363 with slight differences in culture growth rate and color, and 17 nucleotide differences in their TEF-1α sequences. The TEF-1α sequences of *Cadophora vinaceae* CBS 146263 are identical to the ones of our isolates Bent721 and Bent722, and morphological features of Bent721 matched the description of *Cadophora ferruginea* CBS 146363. Accordingly, we considered isolates Bent721 and Bent722 to be *Cadophora ferruginea* based on the present phylogeny of the genus, which further suggests that *Cadophora vinaceae* is invalid and should be synonymized with *Cadophora ferruginea*. Furthermore, three isolates of *Cadophora sabaouae* identified from grapevines in Algeria clustered with strong support (100/100%) with eight isolates of the well-known species *Cadophora luteo-olivacea*, and these three isolates were placed on a branch internal to the one of the type specimen for this species (CBS 141.41). *Cadophora sabaouae* CBS 147192 was described as phylogenetically related to *Cadophora luteo-olivacea* with differences in culture growth rate and 14 nucleotide differences in an alignment of more than 1,400 characters. Given the morphological variability of this species in culture (Harrington and Mcnew, 2003 Gramaje et al., 2011) and the minimal amount of nucleotide differences between sequences of the two species, our phylogenetic results strongly suggest that ‘*Cadophora sabaouae*’ is an internally nested clade that is actually *Cadophora luteo-olivacea*. If not, the clade containing *Cadophora luteo-olivacea* is polyphyletic, which does not conform to the principles of cladistics and monophyly. Therefore, the name *Cadophora sabaouae* is reduced to synonymy with *Cadophora luteo-olivacea*.

*Kalmusia variispora* was isolated from diseased grapevines in Washington and California. The pathogenicity of this species to grape is reported by Abed-Ashtiani et al. (2019), who fulfilled Koch’s postulates and reproduced wood discolorations and wilting symptoms in controlled inoculations of grapevines. In our pathogenicity test, this species was the most virulent among 10 species evaluated. This is the first report of *Kalmusia variispora* in North America. Members of the family Didymosphaeriaceae include plant endophytes, saprobes and pathogens (Ariyawansa et al., 2014), and *Kalmusia variispora* is known as a causal agent of apple fruit core rot (Ntasiou et al., 2021; Gutierrez et al., 2022). Its sister species *Kalmusia longispora* has been shown to cause vascular necrosis in grapevines (Karácsony et al., 2021). Considering *Kalmusia variispora* produces a wide array of phytotoxic metabolites (Cimmino et al., 2021), this species may be associated with other overlooked symptoms.

The charcoal oak decline fungus *Biscogniauxia mediterranea* is a well-known wood pathogen around the world, but has not been reported as a grapevine trunk pathogen, although it is reported from *Vitis* sp. in the US (Jayawardena et al., 2018). The closely related species *Biscogniauxia rosacearum* has been shown pathogenic to grapevine in Iran (Bahmani et al., 2021), and is also known to cause charcoal cankers of pear, plum and quince trees in Italy (Raimondo et al., 2016). *Biscogniauxia mediterranea* isolate Kern007 proved pathogenic in our pathogenicity test. Our findings support the role of *Biscogniauxia mediterranea* as a grapevine trunk pathogen in California.

Members of the genus *Thyrostroma* (Pleosporales, Pleosporineae) frequently are plant pathogens, causing wood cankers, dieback and leaf spots on numerous hosts, with, for example, *Thyrostroma celtidis* and *Thyrostroma lycii* associated with twig cankers of *Celtidis occidentalis* and *Lycium barbarum*, respectively (Senwanna et al., 2019). There are only 14 species in this genus that are currently supported with molecular data, with these species known only from Korea, Russia, Ukraine, USA and Uzbekistan (Hyde et al., 2020). One isolate of *Thyrostroma* (Bent904) was recovered from a wood canker of a *Vitis vinifera* ‘Chardonnay’ vine in Washington. We generated and deposited in GenBank sequences for six loci for this isolate and multilocus phylogenetic analyses revealed this isolate constituted a new phylogenetic species (data not shown) whose closest relative was *Thyrostroma ephedricola* (Pem et al., 2019). We did not formally described our isolate as a new species as we could not obtain, despite multiple attempts, fruiting bodies in culture. Our pathogenicity test revealed *Thyrostroma* sp. Bent904 as pathogenic to grapevine.

In assessing the optimal mycelium growth temperature of 10 isolates from each region, an important finding was the sharp contrast in their growth ability at the extremes of the temperature ranges tested. Most isolates from Washington grew at 5°C, but not at 35°C, whereas most isolates from California did not grow at 5°C, but grew at 35°C. In addition, differences in optimal growth temperatures were observed for isolates from each region. Our findings suggest that the differences in species composition in each region (with only six species shared between regions), may be influenced by local environmental conditions. Although these findings are based on a subset of isolates from each region, they suggest that Washington isolates seem, on average, better adapted to the cooler winter / spring climate found in this region, compared to Californian isolates adaptations to warmer winter / spring climates. Considering that some evidence points to a latitudinal, geographical range shift of plant pests and pathogens influenced by climate change (Bebber, 2015), and that some trunk pathogens are often found in grapevine planting material (Carbone et al., 2022) that is commonly transported across viticulture regions, such future shift in geographical ranges of grapevine trunk pathogens might be expected.

In conclusion, this study illustrates the differences in community composition that can be observed in grapevines expressing similar symptoms of trunk diseases from distinct grape-growing regions and production systems, with climate adaptation a plausible driver of pathogen distributions. By establishing a physical fungal collection, the characterization of the thermophilic profiles and pathogenic status of some isolates could be achieved, expanding the list of fungal pathogens associated with grapevine trunk diseases. Moreover, this study introduced four new fungal species in important genera of plant pathogens. Accurate taxonomic identification coupled with the evaluation of life history traits are essential to understanding the basic ecology and management of individual species, which will undeniably assist into the interpretation of fungal community sequencing datasets.

## Data availability statement

The molecular data presented in this study can be found at the National Institute of Health, genetic sequence database GenBank (https://www.ncbi.nlm.nih.gov/genbank/), with accession numbers provided in **Table 1**. Dried specimens of newly described taxa were deposited at the U.S. National Fungus Collections (BPI) under the following accession numbers: BPI 911228 - BPI 911231 and can be accessed at the herbarium specimen database (https://nt.ars-grin.gov/fungaldatabases/specimens/specimens.cfm). Living cultures of newly described taxa and additional strains presented in **Table 1** have been deposited at the Westerdijk Fungal Biodiversity Institute (https://wi.knaw.nl/) with accession numbers CBS 149294-CBS 149301, CBS 149336 and CBS 149338. Further queries can be directed to the corresponding author.

## Author contributions

RT, MM and KB obtained the funding and designed the study. MM and KB directed the liaison with industry for vineyard selection. DL, PTF and RT contributed to the analyses and interpretation of genetic data. DL conducted phylogenetic analyses. PF and RT guided the acquisition of molecular data. RT and KB conducted the acquisition and interpretation of the pathogenicity data. RT conducted the acquisition and interpretation of the morphological and temperature data. RT wrote the manuscript with contributions from DL, MM and KB. All authors reviewed the manuscript.

## Funding

Funding was provided by the USDA Specialty Crop Multi-State Program, grant 17-0728-001-SF. Additional funding was provided by USDA National Institute of Food and Agriculture, Hatch project 1016563.

## Acknowledgments

The authors would like to express their gratitude to Gabriel A. Torres (University of California Cooperative Extension) for his help in identifying table grape vineyards. The assistance of local grape growers was very much appreciated. Authors thank Maria Mireles (Washington State University) for her help with plant sample acquisition. We also thank Paula J. Eschen and Alejandro I. Hernandez (UC Davis) for assistance with generating molecular sequences and microscopy. Mention of trade names or commercial products is solely for the purpose of providing specific information and does not imply recommendation or endorsement by the USDA. USDA is an equal opportunity provider and employer.

## Conflict of interest

The authors declare that the research was conducted in the absence of any commercial or financial relationships that could be construed as a potential conflict of interest.

## Publisher’s note

All claims expressed in this article are solely those of the authors and do not necessarily represent those of their affiliated organizations, or those of the publisher, the editors and the reviewers. Any product that may be evaluated in this article, or claim that may be made by its manufacturer, is not guaranteed or endorsed by the publisher.
