# Supplementary material for: Fungal species associated with grapevine trunk diseases in Washington wine grapes and California table grapes, with novelties in the genera Cadophora, Cytospora, and Sporocadus
Source: Front Fungal Biol. 2022 Oct 7;3:1018140. doi: 10.3389/ffunb.2022.1018140 (PMC10512239; doi:10.3389/ffunb.2022.1018140)
Supplement: Supplementary file 1 [file DataSheet_1.docx]

Supplementary Material

**Supplementary Table 1. Details of the PCR conditions and primers used for amplification of the six loci used for molecular identification of the isolates recovered from grapevine wood in this study.**

| Locus | Primer Pair | Reference^5^ | Mg (mM) | GoTaq® Flexi Taq (unit) | Initial denaturation (°C) | Initial denaturation (min) | Number of PCR cycles | Denaturation (°C) | Denaturation (s) | Annealing temperature (°C) | Annealing time (s) | Extension time (s) | Final extension time (min) |
| --- | --- | --- | --- | --- | --- | --- | --- | --- | --- | --- | --- | --- | --- |
| ITS | ITS5/ITS4 | White et al. 1990 | 2 | 1 | 95 | 2 | 35 | 95 | 60 | 54 | 60 | 90 | 5 |
| ITS | ITS1/ITS4 | White et al. 1990 | 2 | 1 | 95 | 2 | 35 | 95 | 60 | 54 | 60 | 90 | 5 |
| LSU | LR0R/‌Un‑Lo28S1220 | Bunyard et al. 1996 / ‌Bala et al. 2010 | 2 | 1 | 94 | 5 | 35 | 94 | 60 | 50 | 30 | 20 | 5 |
| LSU | LROR/‌Un‑Lo28S1220 | Rehner & Samuels 1994 / ‌Bala et al. 2010 | 2 | 1 | 94 | 5 | 35 | 94 | 60 | 55 | 50 | 120 | 7 |
| ACT | ACT547F/‌ACT768R^1^ | Baumgartner et al. 2013 | 4 | 1.25 | 95 | 2 | 40 | 94 | 30 | 55 | 30 | 60 | 5 |
| ACT | ACT512F/‌ACT783R | Carbone & Kohn 1999 | 4 | 1.25 | 95 | 2 | 35 | 95 | 60 | 58-64 | 60^6^ | 120^6^ | 5 |
| RPB2 | Fv-RPB2-6F/‌Fv‑RPB2-7R^2^ | this study | 2 | 1 | 94 | 5 | 35 | 94 | 60 | 59 | 60 | 90 | 5 |
| RPB2 | bRPB2-6f/‌bRPB2-7R^3^ | Matheny 2005 | 2 | 1 | 94 | 5 | 35 | 94 | 60 | 59 | 60 | 90 | 5 |
| RPB2 | RPB2-5F2/‌fRPB2‑7cR | Sung et al. 2007/‌Liu et al. 1999 | 2 | 1 | 94 | 5 | 35 | 94 | 60 | 55-58 | 50 | 120 | 7 |
| TEF-1α | EF1-983F/‌EF1‑1567R^4^ | Rehner & Buckley 2005 | 2 | 1 | 94 | 5 | 35 | 94 | 60 | 58 | 30 | 20 | 5 |
| TEF-1α | EF1-728F/‌EF1‑986R | Carbone & Kohn 1999 | 4 | 1.25 | 95 | 2 | 35 | 95 | 60 | 55-58 | 60 | 60 | 5 |
| TEF-1α | EF1-688F/‌EF1‑1251R | Alves et al. 2008 | 4 | 1-1.25 | 95 | 5 | 30 | 94 | 30 | 55 | 45 | 90 | 10 |
| TUB | T1/Bt2b | O’Donnell & Cigelnik 1997/‌Glass & Donaldson 1995 | 2 | 1 | 95 | 2 | 45 | 95 | 60 | 58 | 60 | 120 | 5 |
| TUB | Bt2a/Bt2b | Glass & Donaldson 1995 | 4 | 1.25 | 95 | 2 | 35-36 | 95 | 60 | 58-64 | 60 | 120 | 5 |

^1^ For use with isolates of the genus *Diaporthe*.

^2^ For use with isolates of the genus *Flammulina*. Fv-RPB2-6F: 5’-TGGGGGATGGTTTGTCCAGC-3’; ‌Fv‑RPB2-7R2: 5’- ACTTGRTTATGGTCCGGGAAGGG-3’.

^3^ For use with isolates of the phylum Basidiomycota.

^4^ For use with isolates of the phylum Basidiomycota.

5 References: White, T. J., Bruns, T., Lee, S. and Taylor, J. (1990) Amplification and direct sequencing of fungal ribosomal RNA genes for phylogenetics. In: PCR protocols: A guide to methods and applications. (Innis, M. A., Et Al., ed.). New York: Academic Press Inc., pp. 315-322. Bunyard, B. A., Chaichuchote, S., Nicholson, M. S. and Royse, D. J. (1996) Ribosomal DNA analysis for resolution of genotypic classes of Pleurotus. Mycological Research, 100, 143-150. Bala, K., Robideau, G., Désaulniers, N., De Cock, A. and Lévesque, C. (2010) Taxonomy, DNA barcoding and phylogeny of three new species of Pythium from Canada. Persoonia-Molecular Phylogeny and Evolution of Fungi, 25, 22-31. Rehner, S. A. and Samuels, G. J. (1994) Taxonomy and phylogeny of Gliocladium analysed from nuclear large subunit ribosomal DNA sequences. Mycological Research, 98, 625-634. Baumgartner, K., Fujiyoshi, P. T., Travadon, R., Castlebury, L. A., Wilcox, W. F. and Rolshausen, P. E. (2013) Characterization of species of Diaporthe from wood cankers of grape in eastern North American vineyards. Plant Disease, 97, 912-920. Carbone, I. and Kohn, L. M. (1999) A method for designing primer sets for speciation studies in filamentous ascomycetes. Mycologia, 91, 553-556. Matheny, P. B. (2005) Improving phylogenetic inference of mushrooms with RPB1 and RPB2 nucleotide sequences (Inocybe; Agaricales). Molecular Phylogenetics and Evolution, 35, 1-20. Sung, G.-H., Sung, J.-M., Hywel-Jones, N. L. and Spatafora, J. W. (2007) A multi-gene phylogeny of Clavicipitaceae (Ascomycota, Fungi): Identification of localized incongruence using a combinational bootstrap approach. Molecular Phylogenetics and Evolution, 44, 1204-1223. Liu, Y. J., Whelen, S. and Hall, B. D. (1999) Phylogenetic relationships among ascomycetes: evidence from an RNA polymerse II subunit. Molecular Biology and Evolution, 16, 1799-1808. Rehner, S. A. and Buckley, E. (2005) A Beauveria phylogeny inferred from nuclear ITS and EF1-α sequences: evidence for cryptic diversification and links to Cordyceps teleomorphs. Mycologia, 97, 84-98. Alves, A., Crous, P. W., Correia, A. and Phillips, A. (2008) Morphological and molecular data reveal cryptic speciation in Lasiodiplodia theobromae. Fungal Diversity, 28, 1-13. O'Donnell, K. and Cigelnik, E. (1997) Two divergent intragenomic rDNA ITS2 types within a monophyletic lineage of the fungus Fusarium are nonorthologous. Molecular Phylogenetics and Evolution, 7, 103-116. Glass, N. L. and Donaldson, G. C. (1995) Development of primer sets designed for use with the PCR to amplify conserved genes from filamentous ascomycetes. Applied and Environmental Microbiology, 61, 1323-1330.

^6^ The combined annealing and extension time were in some cases as short as 15s to increase reaction specificity.

**Supplementary Table 2. Details of the isolates and associated GenBank accession numbers included in the phylogenetic analyses of the genus *Cadophora*.**

| Species | Isolate no.^1^ | Host/ substrate | Country | ITS ^2^ | *TUB ^2^* | *EF1-α ^2^* |
| --- | --- | --- | --- | --- | --- | --- |
| *Cadophora africana* | CBS 120890^T^ | *Prunus salicina*, necrotic wood | South Africa | MN232936 | MN232967 | MN232988 |
| *Cadophora antarctica* | CBS 143035^T^ | diesel-contaminated soil sample | Antarctica | MG385664 | – | – |
| *Cadophora bubakii* (as *Phialophora bubakii*) | CBS 198.30 | margarine | Czech Republic | MH855111 | – | MN232989 |
| *Cadophora constrictospora* | CBS 146371^T^ | *Microthlaspi sp*., asymptomatic roots | Bulgaria | KT269023 | – | MN325874 |
| *Cadophora dextrinospora* | CBS 401.78^T^ (=STE-U 5093) | unknown, decaying wood | Spain | NR_119489 | – | – |
| *Cadophora dextrinospora* | AG5 | *Populus tremula* wood colonized by longhorned beetles | Finland | MF188986 | – | – |
| *Cadophora dextrinospora* | ICMP 18083 | *Actinidia deliciosa* wood | New Zealand | HM116746 | – | – |
| *Cadophora domestica* | CBS 146265^T^ | *Prunus domestica*, necrotic tissues at crown | South Africa | MN873024 | MN873028 | MN873031 |
| *Leptodophora echinata* (as *Cadophora echinata*) | CBS 146383^T^ (=P6045) | *Microthlaspi perfoliatum*, asymptomatic root | Spain | KT270239 | – | MN325932 |
| *Cadophora fascicularis* | CBS 146382^T^ | *Microthlaspi perfoliatum*, asymptomatic root | Germany | KT269992 | – | MN325918 |
| *Cadophora fastigiata* | CBS 307.49 | *Pinus* sp., blue stain | Sweden | MH856538 | KM497131 | KM497087 |
| *Cadophora ferruginea* | CBS 146363^T^ (=P1323) | *Microthlaspi perfoliatum*, asymptomatic root | Spain | KT268618 | – | MN325861 |
| *Leptodophora gamsii* (as *Cadophora gamsii*) | CBS 146379^T^ (=P2437) | *Microthlaspi perfoliatum*, asymptomatic root | France | KT269668 | – | MN325899 |
| *Cadophora gregata* | ATCC 11073^T^ | *Glycine max*, brown stem rot | Japan | U66731 | MF677920 | MF979586 |
| *Cadophora helianthi* | CBS 144752^T^ | *Helianthus annuus*, necrotic tissue in stem | Ukraine | MF962601 | MH733391 | MH719029 |
| *Cadophora interclivum* | CBS 143323^T^ | *Carex sprengelii*, root | Canada | MF979577 | MF677917 | MF979583 |
| *Cadophora interclivum* | BAP33 | *Picea glauca*, root | Canada | MF979578 | MF677918 | MF979584 |
| *Cadophora lacrimiformis* | MFLU 16-1486^T^ | unknown Brassicaceae, dead stem | Russia | MK585003 | – | – |
| *Cadophora luteo-olivacea* | A19 | *Vitis vinifera*, vascular discoloration | USA | KM497038 | KM497119 | KM497075 |
| *Cadophora luteo-olivacea* | A41 | *Vitis vinifera* 'Chardonnay', vascular discoloration | USA | KM497039 | KM497120 | KM497076 |
| *Cadophora luteo-olivacea* | A42 | *Vitis vinifera* 'Chardonnay', vascular discoloration | USA | KM497040 | KM497121 | KM497077 |
| *Cadophora luteo-olivacea* | CBS 141.41^T^ (=A175) | waste water | Sweden | MH856538 | KM497131 | KM497087 |
| *Cadophora luteo-olivacea* | CBS 357.51 | *Malus sylvestris*, fruit | Italy | AY249068 | – | KF764611 |
| *Cadophora luteo-olivacea* | GLMC 517 | *Prunus domestica*, necrotic wood | Germany | MN232937 | MN232968 | MN233003 |
| *Cadophora luteo-olivacea* | CBS 145525 (=GLMC 1310) | *Prunus domestica*, necrotic wood | Germany | MN232939 | MN232970 | MN233005 |
| *Cadophora luteo-olivacea* | U17 | *Vitis vinifera* 'Chardonnay', vascular discoloration | USA | KM497043 | KM497124 | KM497080 |
| *Cadophora malorum* | CBS 165.42^T^ | *Amblystoma mexicanum* | The Netherlands | AY249059 | KM497134 | KM497090 |
| *Cadophora margaritata* | CBS 144083^T^ | *Populus tremula*, wood colonized by *Saperda carcharias* | Finland | KJ702027 | MH327786 | – |
| *Cadophora melinii* | CBS 268.33^T^ (=A164) | probably wood-pulp | Sweden | NR111150 | KM497132 | KM497088 |
| *Cadophora melinii* | ONC1 | *Vitis vinifera* 'Cabernet Franc', wood canker | Canada | KM497033 | KM497114 | KM497070 |
| *Cadophora meredithiae* | CBS 143322^T^ | *Carex sprengelii*, root | Canada | MF979574 | MF677914 | MF979580 |
| *Cadophora meredithiae* | BAP6 | *Picea glauca*, root | Canada | MF979575 | MF677915 | MF979581 |
| *Cadophora microspora* | MFLU 18-2672^T^ | unknown Apiaceae, stem | UK | MK584939 | – | – |
| *Cadophora novi-eboraci* | CBS 145757 (=GLMC 688) | *Prunus cerasus*, necrotic wood | Germany | MN232946 | MN232977 | MN232994 |
| *Cadophora novi-eboraci* | NYC14^T^ | *Vitis labruscana*, wood canker | USA | KM497037 | KM497118 | KM497074 |
| *Cadophora obovata* | CBS 146374^T^ | *Microthlaspi perfoliatum*, asymptomatic root | Germany | KT269230 | – | MN325888 |
| *Cadophora obscura* (as *Phialophora bubakii*) | CBS 269.33 | fresh water | Sweden | MN232948 | – | MN232996 |
| *Leptodophora orchidicola* (as *Cadophora orchidicola*) | UAMH8152 | *Pedicularis bracteosa*, root | Canada | AF214576 | MF677921 | MF979587 |
| *Cadophora orientoamericana* | NHC1^T^ (=MYA-4972) | *Vitis labrusca* 'Niagara', wood canker | USA | KM497018 | KM497099 | KM497055 |
| *Cadophora orientoamericana* | NYC12 | *Vitis vinifera* 'Chardonnay', wood canker | USA | KM497025 | KM497106 | KM497062 |
| *Cadophora prunicola* | CBS 120891^T^ | *Prunus salicina*, necrotic wood | South Africa | MN232949 | MN232979 | MN232997 |
| *Cadophora prunicola* | GLMC 1574 | *Prunus domestica*, necrotic wood | Germany | MN232954 | MN232982 | MN233000 |
| *Cadophora ramosa* | CBS 145523^T^ (=GLMC 377) | *Prunus cerasus*, necrotic wood | Germany | MN232956 | MN232984 | MN233002 |
| *Cadophora ramosa* (as *C. spadicis*) | CBS 111743 | *Actinidia chinensis*, vascular discoloration | Italy | DQ404351 | KM497136 | KM497091 |
| *Cadophora ramosa* (as *C. spadicis*) | QCC1 | *Vitis vinifera* 'Gamay', wood canker | Canada | KM497031 | KM497112 | KM497068 |
| *Cadophora ramosa* (as *C. spadicis*) | RIC1 | *Vitis vinifera* 'Cabernet Sauvignon', wood canker | USA | KM497029 | KM497110 | KM497066 |
| *Cadophora ramosa* (as *C. spadicis*) | RIC3 | *Vitis* hybrid 'Vidal', wood canker | USA | KM497030 | KM497111 | KM497067 |
| *Cadophora rotunda* | CBS 146264^T^ | *Prunus domestica*, necrotic tissues at crown | South Africa | MN873023 | MN873029 | MN873030 |
| *Cadophora luteo-olivaceae (as C. sabaouae)* | CBS 147192^T^ (=WAMC34) | *Vitis vinifera* | Algeria | MT644187 | MT646749 | MT646746 |
| *Cadophora luteo-olivaceae (as C. sabaouae)* | WAMC117 | *Vitis vinifera* | Algeria | MT524745 | MT646750 | MT646747 |
| *Cadophora luteo-olivaceae (as C. sabaouae)* | WAMC118 | *Vitis vinifera* | Algeria | MT524744 | MT646751 | MT646748 |
| *Leptodophora variabilis* (as *Cadophora variabilis*) | CBS 146360^T^ (=P1176) | *Microthlaspi perfoliatum*, asymptomatic root | Croatia | KT268493 | – | MK550890 |
| *Cadophora vinacea* | CBS 146263^T^ | *Vitis vinifera* 'Ehrenfelser', necrotic wood | Canada | MN873025 | MN873027 | MN873032 |
| *Cadophora viticola* | CBS 139517^T^ (=Cme-2) | *Vitis vinifera* 'Syrah', black streaks in shoots | Spain | HQ661097 | HQ661067 | HQ661082 |
| *Cadophora vivarii* | CBS 146262^T^ | *Malus domestica*, necrotic tissues at bud union | South Africa | KY312633 | MN873026 | MN873033 |
| *Hyaloscypha finlandica* | CBS 444.86^T^ | *Pinus sylvestris*, root of seedling | Finland | AF486119 | KM497130 | KM497086 |
| *Hyaloscypha finlandica* | IFM 50530 | NA | NA | AB190393 | – | – |

^1^ ATCC: American Type Culture Collection, Manassas, USA; CBS: Culture collection of the Westerdijk Fungal Biodiversity Institute, Utrecht, The Netherlands; GLMC: Culture collection of Senckenberg Museum of Natural History Görlitz, Görlitz, Germany; ICMP: International Collection of Microorganisms from Plants, Auckland, New Zealand; MFLU: Mae Fah Luang University, Chiang Rai, Thailand; STEU: University of Stellenbosch, Stellenbosch, South Africa; UAMH: Centre for Global Microfungal Biodiversity, Toronto, Canada.

^2^ITS: internal transcribed spacers and intervening 5.8S nrDNA; TUB: β-tubulin gene; EF1-α: translation elongation factor 1-α gene.

^T^ex-type cultures

NA: not available.

**Supplementary Table 3. Details of the isolates and associated GenBank accession numbers included in the phylogenetic analyses of the genus *Cytospora*.**

| Species | Isolate no.^1^ | Host | Origin | ITS^2^ | LSU^2^ | *ACT^2^* | *RPB2^2^* | *TEF1-α^2^* | *TUB2^2^* |
| --- | --- | --- | --- | --- | --- | --- | --- | --- | --- |
| *C. ailanthicola* | CFCC 89970^T^ | *Ailanthus altissima* | Ningxia, China | MH933618 | MH933653 | MH933526 | MH933592 | MH933494 | MH933565 |
| *C. ampulliformis* | MFLUCC 16-0583^T^ | *Sorbus intermedia* | Russia | KY417726 | KY417760 | KY417692 | KY417794 | NA | NA |
| *C. ampulliformis* | MFLUCC 16-0629 | *Acer platanoides* | Russia | KY417727 | KY417761 | KY417693 | KY417795 | NA | NA |
| *C. chrysosperma* | CFCC 89629 | *Salix psammophila* | Shaanxi, China | KF765673 | KF765689 | NA | KF765705 | NA | NA |
| *C. chrysosperma* | CFCC 89981 | *Populus alba* subsp. *pyramidalis* | Gansu, China | MH933625 | MH933660 | MH933533 | MH933597 | MH933501 | MH933568 |
| *C. chrysosperma* | CFCC 89982 | *Ulmus pumila* | Tibet, China | KP281261 | KP310805 | KP310835 | KU710952 | KP310848 | KP310818 |
| *C. cotini* | MFLUCC 14-1050^T^ | *Cotinus coggygria* | Russia | KX430142 | KX430143 | NA | KX430144 | NA | NA |
| *C. fraxiicola* | MFLU 17-2392 | dead branches | Russia | NA | MN764356 | MN995562 | NA | NA | NA |
| *C. galegicola* | MFLUCC 18-1199^T^ | *Galega officinalis* | Forlì-Cesena, Italy | MK912128 | MK571756 | MN685810 | MN685820 | NA | NA |
| *C. joaquinensis* | CBS 144235^T^ | *Populus deltoides* | California, USA | MG971895 | NA | MG972044 | NA | MG971605 | **OP079911** |
| *C. joaquinensis* | 9E-95 | *Juglans regia* | California, USA | MG971896 | NA | MG972045 | NA | MG971606 | **OP079912** |
| *C. joaquinensis* | 9E-44 | *Pistacia vera* | California, USA | MG971897 | NA | MG972046 | NA | MG971607 | **OP079913** |
| *C. joaquinensis* | KARE195 | *Pistacia vera* | California, USA | MG971894 | NA | MG972043 | NA | MG971604 | **OP079914** |
| *C. joaquinensis* | KARE231 | *Pistacia vera* | California, USA | MG971893 | NA | MG972042 | NA | MG971603 | **OP079915** |
| *C. longiostiolata* | MFLUCC 16-0628^T^ | *Salix* × *fragilis* | Russia | KY417734 | KY417768 | KY417700 | KY417802 | NA | NA |
| *C. longispora* | CBS 144236^T^ | *Prunus domestica* | California, USA | MG971905 | NA | MG972054 | NA | MG971615 | **OP079916** |
| *C. melnikii* | CFCC 89984 | *Rhus typhina* | Xinjiang, China | MH933644 | MH933678 | MH933551 | MH933609 | MH933515 | MH933580 |
| *C. melnikii* | MFLUCC 15-0851^T^ | *Malus domestica* | Russia | KY417735 | KY417769 | KY417701 | KY417803 | NA | NA |
| *C. melnikii* | MFLUCC 16-0635 | *Populus nigra* var. *italica* | Russia | KY417736 | KY417770 | KY417702 | KY417804 | NA | NA |
| *C. platycladicola* | CFCC 50038^T*^ | *Platycladus orientalis* | Gansu, China | KT222840 | MH933682 | MH933555 | MH933613 | MH933519 | MH933584 |
| *C. platycladicola* | CFCC 50039* | *Platycladus orientalis* | Gansu, China | KR045642 | KR045721 | KU711008 | KU710973 | KU710931 | KR045683 |
| *C. populicola* | CBS 144240^T^ | *Populus deltoides* | California, USA | MG971891 | NA | MG972040 | NA | MG971601 | **MG971757** |
| *C. prunicola* | MFLUCC 18-1200 | *Quercus pubescens* | Italy | MK912129 | MK571757 | MN685811 | NA | NA | NA |
| *C. prunicola* | MFLU 17-0995^T^ | *Prunus sp.* | Italy | MG742350 | MG742351 | MG742353 | MG742352 | MG742354 | NA |
| *C. ribis* | CFCC 50026^T^ | *Ulmus pumila* | Qinghai, China | KP281267 | KP310813 | KP310843 | KU710972 | KP310856 | KP310826 |
| *C. ribis* | CFCC 50027 | *Ulmus pumila* | Qinghai, China | KP281268 | KP310814 | KP310844 | NA | KP310857 | KP310827 |
| *C. rosigena* | MFLUCC 18-0921^T^ | *Rosa* sp. | Russia | MN879872 | MN879873 | NA | NA | NA | NA |
| *C. rostrata* | CFCC 89909^T^ | *Salix cupularis* | Gansu, China | KR045643 | KR045722 | KU711009 | KU710974 | KU710932 | KR045684 |
| *C. rostrata* | CFCC 89915 | *Salix cupularis* | Gansu, China | KR045644 | KR045723 | KU711010 | KU710975 | KU710933 | KR045685 |
| *C. salicacearum* | MFLUCC 16-0576 | *Populus nigra* var. *italica* | Russia | KY417747 | KY417781 | KY417713 | KY417815 | NA | NA |
| *C. salicacearum* | MFLUCC 16-0587 | *Prunus cerasus* | Russia | KY417748 | KY417782 | KY417714 | KY417816 | NA | NA |
| *C. salicacearum* | MFLUCC 15-0861 | *Salix* × *fragilis* | Russia | KY417745 | KY417779 | KY417711 | KY417813 | NA | NA |
| *C. salicacearum* | MFLUCC 15-0509^T^ | *Salix alba* | Russia | KY417746 | KY417780 | KY417712 | KY417814 | NA | NA |
| *C. salicina* | MFLUCC 15-0862^T^ | *Salix alba* | Russia | KY417750 | KY417784 | KY417716 | KY417818 | NA | NA |
| *C. salicina* | MFLUCC 16-0637 | *Salix* × *fragilis* | Russia | KY417751 | KY417785 | KY417717 | KY417819 | NA | NA |
| *C. sophoriopsis* | CFCC 89600^T^ | *Styphnolobium japonicum* | Gansu, China | KR045623 | KP310804 | KU710992 | KU710951 | KU710915 | KP310817 |
| *C. tanaitica* | MFLUCC 14-1057^T^ | *Betula pubescens* | Russia | KT459411 | KT459412 | KT459413 | NA | NA | NA |
| *C. ulmi* | MFLUCC 15-0863^T^ | *Ulmus minor* | Russia | KY417759 | KY417793 | KY417725 | KY417827 | NA | NA |
| *C. ulmicola* | MFLUCC 18-1227^T^ | *Ulmus pumila* | Russia | MH940220 | MH940218 | MH940216 | NA | NA | MH986792 |
| *C. viticola* | Cyt2 | *Vitis* hybrid ‘Frontenac’ | USA | KX256238 | NA | NA | NA | KX256259 | **OP079917** |
| *C. viticola* | CBS 141586^T^ | *Vitis vinifera* ‘Cabernet franc’ | USA | KX256239 | NA | NA | NA | KX256260 | **OP079910** |
| *D. ampelina* | CBS 114016 | *Vitis vinifera* | South Africa | AF230751 | NA | JQ807296 | NA | GQ250351 | JX275452 |
| *D. ampelina* | Wolf912 | *Vitis vinifera* | California, USA | KM669964 | NA | JGI^3^ | JGI^3^ | KM669820 | JGI^3^ |

^1^ CBS: Culture collection of the Westerdijk Fungal Biodiversity Institute, Utrecht, The Netherlands; CFCC: China Forestry Culture Collection Centre, Beijing, China; KARE: Kearney Agricultural Research and Extension Center, Kearney, USA; MFLU: Mae Fah Luang University, Chiang Rai, Thailand; MFLUCC: Mae Fah Luang University Culture Collection, Thailand. ^T^ex-type cultures.

^2^ITS: internal transcribed spacers and intervening 5.8S nrDNA; LSU: nuclear large subunit ribosomal DNA; ACT: actin; RPB2: second largest subunit of RNA polymerase II; EF1-α: translation elongation factor 1-α gene; TUB2: β-tubulin gene. Sequences in bold were generated in this study.

^3^JGI: sequences retrieved from Joint Genome Institute Mycocosm genome portal.

NA: not available.

**Supplementary Table 4. Details of the isolates and associated GenBank accession numbers included in the phylogenetic analyses of the genus *Sporocadus*.**

| Species | Isolate no.^1^ | Host / substrate | Country | LSU^2^ | ITS^2^ | TUB^2^ | TEF-1α^2^ | RPB2^2^ |
| --- | --- | --- | --- | --- | --- | --- | --- | --- |
| *Seimatosporium luteosporum* | CBS 142599^T^ (=Wint754) | *Vitis vinifera* | USA | KY706309 | KY706284 | KY706259 | KY706334 | NA |
| *Seimatosporium luteosporum* | Napa754 | *Vitis vinifera* | USA | KY706308 | KY706283 | KY706258 | KY706333 | NA |
| *Seimatosporium pistaciae* | CBS 138865^T^ (=CPC 24455) | *Pistacia vera* | Iran | KP004491 | KP004463 | MH554674 | MH554432 | MH554915 |
| *Sporocadus biseptatus* | CBS 110324^T^ (=MYC 754) | NA | NA | MH554179 | MH553956 | MH554615 | MH554374 | MH554853 |
| *Sporocadus cornicola* | CBS 143889 (=CPC 23235) | *Cornus sanguinea* | Germany | MH554326 | MH554121 | MH554794 | MH554555 | MH555029 |
| *Sporocadus cornicola* | MFLUCC 14-0448^T^ | *Cornus sanguinea* | Italy | NA | KU974967 | NA | NA | NA |
| *Sporocadus cornii* | MFLUCC 14-0467^T^ | *Cornus sp.* | Italy | KR559739 | KT162918 | NA | NA | NA |
| *Sporocadus glandigenum* | NBRC 32677 | *Fagus sylvatica* | Japan | AB593735 | AB594803 | NA | NA | NA |
| *Sporocadus cotini* | CBS 139966^T^ (=MFLUCC 14-0623) | *Cotinus coggygria* | Russia | MH554222 | MH554003 | MH554675 | MH554433 | MH554916 |
| *Sporocadus incanus* | CBS 123003^T^ | *Prunus dulcis* | Spain | MH554210 | MH553991 | MH554659 | MH554417 | MH554900 |
| *Sporocadus kurdistanicus* | CBS 143778^T^ (=IRAN2356C) | *Vitis vinifera* | Iran | MW361958 | MW361950 | MW375350 | MW375356 | NA |
| *Sporocadus kurdistanicus* | IRAN2354C | *Vitis vinifera* | Iran | MW361957 | MW361949 | MW375349 | MW375355 | NA |
| *Sporocadus kurdistanicus* | IRAN2313C | *Vitis vinifera* | Iran | MW361956 | MW361948 | MW375348 | MW375354 | NA |
| *Sporocadus lichenicola* | CBS 354.90 (=NBRC 32677) | *Fagus sylvatica* | Germany | MH554252 | MH554035 | MH554711 | MH554470 | MH554948 |
| *Sporocadus lichenicola* | CPC 24528 | *Juniperus communis* | Germany | MH554332 | MH554127 | MH554800 | MH554562 | MH555036 |
| *Sporocadus lichenicola* | NBRC 32625 (=IMI 079706) | *Rosa canina* | UK | MH883646 | MH883643 | MH883645 | MH883644 | MH883647 |
| *Sporocadus lichenicola* | MFLUCC 14-0052^T^ | *Rosa canina* | Italy | KT005514 | KT005515 | NA | NA | NA |
| *Sporocadus mali* | CBS 446.70^T^ | *Malus sylvestris* | Netherlands | MH554261 | MH554049 | MH554725 | MH554484 | MH554960 |
| *Sporocadus microcyclus* | CBS 424.95^T^ | *Sorbus aria* | Germany | MH554258 | MH554045 | MH554721 | MH554480 | MH554956 |
| *Sporocadus microcyclus* | CBS 887.68 (=NBRC 32680) | *Ribes* sp. | Netherlands | MH554280 | MH554068 | MH554744 | MH554504 | MH554981 |
| *Sporocadus multiseptatus* | CBS 143899^T^ (=CPC 26606) | *Viburnum* sp. | Serbia | MH554343 | MH554141 | MH554814 | MH554576 | MH555047 |
| *Sporocadus pseudorosarum* | MFLUCC 14-0466 | *Rosa canina* | Italy | KT281912 | KT284775 | NA | NA | NA |
| *Sporocadus rosarum* | CBS 113832 (=UPSC 2172) | *Rosa canina* | Sweden | MH554189 | MH553970 | MH554629 | MH554388 | MH554864 |
| *Sporocadus rosarum* | MFLUCC 15-0563^3^ | *Rosa canina* | Italy | MG829071 | MG828960 | NA | NA | NA |
| *Sporocadus rosarum* | MFLUCC 14-0466^3^ | *Rosa canina* | Italy | KT281912 | KT284775 | NA | NA | NA |
| *Sporocadus rosigena* | CBS 116498 | *Vitis vinifera* | Iran | MH554200 | MH553983 | MH554642 | MH554401 | MH554883 |
| *Sporocadus rosigena* | CBS 129166 (=MSCL 860) | *Rhododendron* | Latvia | MH554215 | MH553996 | MH554665 | MH554423 | MH554905 |
| *Sporocadus rosigena* | CBS 182.50 | *Pyrus communis* | Netherlands | MH554233 | MH554013 | MH554689 | MH554447 | MH554926 |
| *Sporocadus rosigena* | CBS 250.49 | *Rubus fruticosus* | Netherlands | MH554245 | MH554023 | MH554699 | MH554457 | MH554934 |
| *Sporocadus rosigena* | CBS 466.96 | Inner tissue of zoocecidium, caused by *Lasioptera rubi*, on *Rubus* sp. | Netherlands | MH554265 | MH554052 | MH554728 | MH554487 | MH554965 |
| *Sporocadus rosigena* | MFLU 16-0239^T^ | *Rosa canina* | Italy | MG829069 | MG828958 | NA | NA | NA |
| *Sporocadus rotundatus* | CBS 616.83^T^ | *Arceuthobium pussilum* | Canada | MH554273 | MH554060 | MH554737 | MH554496 | MH554974 |
| *Sporocadus sorbi* | CBS 160.25 | **–** | **–** | MH554229 | MH554008 | MH554684 | MH554442 | MH554924 |
| *Sporocadus sorbi* | MFLUCC 14-0469^T^ | *Sorbus torminalis* | Italy | KT281911 | KT284774 | NA | NA | NA |
| *Sporocadus sp. 1* | CBS 506.71 | *Euphorbia* sp. | Italy | MH554268 | MH554055 | MH554731 | MH554490 | MH554968 |
| *Sporocadus trimorphus* | CBS 114203^T^ (=UPSC 2430) | *Rosa canina* | Sweden | MH554196 | MH553977 | MH554636 | MH554395 | MH554876 |

^1^ CBS: Culture collection of the Westerdijk Fungal Biodiversity Institute, Utrecht, The Netherlands; CPC: Culture collection of Pedro Crous, housed at the Westerdijk Institute; MFLU: Mae Fah Luang University, Chiang Rai, Thailand; MFLUCC: Mae Fah Luang University Culture Collection, Thailand; MSCL: Microbial Strain Collection of Latvia; NBRC: Biological Resource Center; ^T^ex-type cultures.

^2^ITS: internal transcribed spacers and intervening 5.8S nrDNA; LSU: nuclear large subunit ribosomal DNA; RPB2: second largest subunit of RNA polymerase II; EF1-α: translation elongation factor 1-α gene; TUB2: β-tubulin gene.

^3^MFLUCC 15-0563: Type of *Seimatosporium rosigenum*; MFLUCC 14-0466: Type of *Seimatosporium pseudorosarum*.

NA: not available.

**Supplementary Table 5. Parameters derived from fitting a Gaussian function [f = a*exp(-.5*((x-x0)/b)^2)] to predict the average colony diameter of each isolate based on the dependent variable temperature. Optimal growth temperature was tested by culturing each isolate in triplicate in the dark at temperatures ranging from 5 °C to 35 °C at 5 °C increments in duplicated experiments.**

| Species | Isolate^1^ | *a*^2^ | *b*^3^ | x_0_^4^ | *R*^2 5^ |
| --- | --- | --- | --- | --- | --- |
| *Eutypella citricola* | Kern003 | 60.1239 | 5.6831 | 25.5039 | 0.9363 |
| *Eutypella citricola* | Kern004 | 67.1388 | 6.2814 | 24.7064 | 0.9458 |
| *Diatrypella verruciformis* | Kern006 | 80.2159 | 5.4829 | 21.5422 | 0.9426 |
| *Biscogniauxia mediterranea* | Kern007 | 75.4967 | 8.0556 | 29.7943 | 0.9964 |
| *Phaeoacremonium scolity* | Kern701 | 34.6237 | 5.6651 | 24.6362 | 0.9535 |
| *Phaeoacremonium parasiticum* | Kern706 | 30.5093 | 8.13 | 27.8372 | 0.9991 |
| *Lasiodiplodia gilanensis* | Kern803 | 83.492 | 8.0145 | 27.5572 | 0.9975 |
| *Biscogniauxia mediterranea* | Kern805 | 58.9895 | 6.2603 | 29.0218 | 0.9966 |
| *Diaporthe ampelina* | Kern904 | 67.6388 | 5.4515 | 25.71 | 0.9033 |
| *Cytospora macropycnidia* | Kern907 | 87.0908 | 5.4454 | 20.2929 | 0.9361 |
| *Sporocadus kurdistanicus* | Bent505 | 75.0261 | 5.8655 | 20.7848 | 0.895 |
| *Sporocadus kurdistanicus* | Bent510 | 65.1674 | 6.3943 | 20.3856 | 0.8707 |
| *Diatrype stigma* | Bent015 | 69.6287 | 5.7742 | 23.5067 | 0.9383 |
| *Cadophora columbiana* | Bent717 | 25.6459 | 5.9248 | 20.0702 | 0.9302 |
| *Cadophora columbiana* | Bent718 | 27.7892 | 5.7733 | 20.2436 | 0.8591 |
| *Cadophora ferruginea* | Bent721 | 24.7477 | 7.0381 | 19.2326 | 0.9425 |
| *Cadophora ferruginea* | Bent722 | 25.8199 | 6.8604 | 19.4509 | 0.9339 |
| *Cytospora yakimana* | Bent902 | 73.9673 | 7.5389 | 27.1615 | 0.9618 |
| *Cytospora yakimana* | Bent903 | 71.8041 | 7.5515 | 27.4441 | 0.9722 |
| *Thyrostroma sp.* | Bent904 | 57.2996 | 5.6402 | 16.23 | 0.9621 |

^1^ Kern isolates originate from California and Bent isolates originate from Washington state.

^2^ Parameter *a* is the height of the curve's peak.

^3^ Parameter *b* is the Gaussian root mean square width of the peak.

^4^ Parameter x_0_ is the position of the center of the peak corresponding to the optimal growth temperature (°C) of each isolate.

^5^*R*^2^: coefficient of determination. *R*^2^ equals 0 when the values of the independent variable does not allow any prediction of the dependent variables, and equals 1 when you can perfectly predict the dependent variables from the independent variables.
